# Social buffering diminishes fear response but does not equal improved fear extinction

**DOI:** 10.1093/cercor/bhac395

**Published:** 2022-10-11

**Authors:** Tomasz Gorkiewicz, Konrad Danielewski, Karolina Andraka, Kacper Kondrakiewicz, Ksenia Meyza, Jan Kaminski, Ewelina Knapska

**Affiliations:** Neurobiology of Emotions Laboratory, Nencki-EMBL Partnership for Neural Plasticity and Brain Disorders - BRAINCITY, Nencki Institute of Experimental Biology of Polish Academy of Sciences, 3 Pasteur Street, 02-093 Warsaw, Poland; Neurobiology of Emotions Laboratory, Nencki-EMBL Partnership for Neural Plasticity and Brain Disorders - BRAINCITY, Nencki Institute of Experimental Biology of Polish Academy of Sciences, 3 Pasteur Street, 02-093 Warsaw, Poland; Neurobiology of Emotions Laboratory, Nencki-EMBL Partnership for Neural Plasticity and Brain Disorders - BRAINCITY, Nencki Institute of Experimental Biology of Polish Academy of Sciences, 3 Pasteur Street, 02-093 Warsaw, Poland; Neurobiology of Emotions Laboratory, Nencki-EMBL Partnership for Neural Plasticity and Brain Disorders - BRAINCITY, Nencki Institute of Experimental Biology of Polish Academy of Sciences, 3 Pasteur Street, 02-093 Warsaw, Poland; NeuroElectronics Research Flanders, Leuven, Belgium; Neurobiology of Emotions Laboratory, Nencki-EMBL Partnership for Neural Plasticity and Brain Disorders - BRAINCITY, Nencki Institute of Experimental Biology of Polish Academy of Sciences, 3 Pasteur Street, 02-093 Warsaw, Poland; Neurophysiology of Mind Laboratory, Nencki-EMBL Partnership for Neural Plasticity and Brain Disorders - BRAINCITY, Nencki Institute of Experimental Biology of Polish Academy of Sciences, 3 Pasteur Street, 02-093 Warsaw, Poland; Neurobiology of Emotions Laboratory, Nencki-EMBL Partnership for Neural Plasticity and Brain Disorders - BRAINCITY, Nencki Institute of Experimental Biology of Polish Academy of Sciences, 3 Pasteur Street, 02-093 Warsaw, Poland

**Keywords:** anterior cingulate cortex, fear memory extinction, infralimbic cortex, prelimbic cortex, post-traumatic stress disorders

## Abstract

Social support during exposure-based psychotherapy is believed to diminish fear and improve therapy outcomes. However, some clinical trials challenge that notion. Underlying mechanisms remain unknown, hindering the understanding of benefits and pitfalls of such approach. To study social buffering during fear extinction, we developed a behavioral model in which partner’s presence decreases response to fear-associated stimuli. To identify the neuronal background of this phenomenon, we combined behavioral testing with c-Fos mapping, optogenetics, and chemogenetics. We found that the presence of a partner during fear extinction training causes robust inhibition of freezing; the effect, however, disappears in subjects tested individually on the following day. It is accompanied by lowered activation of the prelimbic (PL) and anterior cingulate (ACC) but not infralimbic (IL) cortex. Accordingly, blocking of IL activity left social buffering intact. Similarly, inhibition of the ventral hippocampus–PL pathway, suppressing fear response after prolonged extinction training, did not diminish the effect. In contrast, inhibition of the ACC–central amygdala pathway, modulating social behavior, blocked social buffering. By reporting that social modulation of fear inhibition is transient and insensitive to manipulation of the fear extinction-related circuits, we show that the mechanisms underlying social buffering during extinction are different from those of individual extinction.

## Introduction

Social groups provide the support that could make exposure-based treatment for post-traumatic stress disorders (PTSD) and phobias more effective. With increased treatment demands, group therapy appears to be more feasible than an individual approach. However, recent analysis of the benefits of group approach points to certain issues (Sloan and Beck 2016). While some studies confirm the benefits of such therapy as compared with no treatment (Beck et al. 2009; Schwartze et al. 2019), the result of a group of randomized clinical trials suggests that treatment administered in an individual format may be more effective (Resick et al. 2017). The mechanisms underlying social influence on exposure therapy remain unknown, which hinders the understanding of benefits and potential pitfalls of the group approach.

Fear extinction is a well-established animal model of exposure therapy. In fear extinction, the conditioned stimulus (CS) previously associated with a threat-inducing stimulation is repeatedly presented without any aversive consequences, through which it loses its predictive value (Quirk 2006). The neuronal mechanisms of fear extinction are relatively well described, with the prefrontal cortex, hippocampus, and basolateral amygdala strongly implicated in the process (Maren 2011). However, we know little about the social buffering impact on fear inhibition.

The studies on social modulation of fear revealed that in social species, distressed individuals show a better recovery from aversive experiences when they have a companion (Thorsteinsson et al. 1998; Kiyokawa et al. 2007, 2014). This phenomenon is called “social buffering.” The presence of a conspecific reduces fear responses to threat (Davitz and Mason 1955; Kiyokawa et al. 2004, 2007; Brill-Maoz and Maroun 2016; Mikami et al. 2016) and the magnitude of the effect depends on the familiarity and emotional status of the partner animal (Kikusui et al. 2006; Hennessy et al. 2009). The presence of a partner reduces fear also during fear extinction (Brill-Maoz and Maroun 2016; Mikami et al. 2016, 2020). However, the role of different social factors during fear extinction and the underlying neural mechanisms of fear inhibition are unknown. Importantly, it has not been investigated whether the impact of social buffering on fear extinction is permanent or transient and whether it improves fear extinction or modulates processes different from fear extinction.

To investigate the role of social buffering in fear extinction, we developed a rat behavioral paradigm in which we tested how context and different social factors, such as the emotional status, familiarity, and strain identity of the partner, modulate the magnitude of social inhibition of fear. Unlike the earlier studies, to model a traumatic experience we used fear conditioning protocol that resulted in a strong fear reaction (Brill-Maoz and Maroun 2016; Penha Farias et al. 2019). Further, we compared the long-term fear memory of subject rats subjected to extinction alone and in the presence of a partner. Next, we identified the neuronal circuits involved in socially supported extinction by combining the behavioral testing with c-Fos mapping, optogenetics, and chemogenetics.

## Materials and methods

### Animals

Experimental subjects were 226 adult, experimentally naïve male Wistar rats (250–300 g at the beginning of the experiment), supplied by the Center of Experimental Medicine in Bialystok, Poland. Additionally, we used the partner rats that did not undergo behavioral analysis, which came from the same source. The animals were randomly paired (at least 2 weeks before the onset of experimental procedures to allow creation of social bonds between cagemates) and housed together in standard polycarbonate home cages (430 × 250 × 185 mm) under a 12/12 light–dark cycle, with food and water provided ad libitum. The floor of the cages was covered with cubic poplar wood granulates (Select, Safe). The rats were habituated to the experimenter’s hand for 2 days and next to the transportation, experimental room and separation for 3 subsequent days prior to the experiment.

All experiments were carried out in accordance with the Polish Act on Animal Welfare, after obtaining specific permission from the First Warsaw Ethical Committee on Animal Research (36/2016).

### Behavioral paradigms

Here we utilized fear conditioning and fear memory extinction protocols (Kondrakiewicz et al. 2019). Before the onset of the experiment, rats were habituated to the experimenter’s hand and the experimental room. In each pair of rats, one individual was marked as a subject and the other as a partner. In this behavioral paradigm, 2 distinct contexts were used:

**Context A** (for conditioning): room lights on, the cage cleaned with a 1% ammonium solution, the rats are transported to this context in transparent plastic boxes on a cart.

**Context B** (for fear extinction and test): room lights off, with 60 W red light on; the cage cleaned with a 1% acetic acid solution, the rats transported to this context in black plastic boxes by hand.

The entire behavioral experiment was conducted in the Panlab shuttle-box for rats (LE916), which consists of 2 equally sized compartments (590 (W) x 190 (D) x 240 (H) each) made with methacrylate and equipped with 2 independent grid floors and speakers located on the top of side walls. Chambers were divided with a perforated transparent partition (wire mesh) allowing the rats to see, hear and smell the neighbor, but not to experience a full physical contact.

The behavioral paradigm lasted 5 consecutive days:

**Day 1.** Conditioning to an auditory CS (in context A). Both rats: subject and partner were subjected to fear conditioning separately in different parts of the experimental chamber. After initial 2 min of habituation, they received 5 CS (pure tones, 20 s duration, 85 dB, 2 kHz) co-terminated with a US (footshock, 1 s duration, 0.7 mA). Subsequent CS’s were separated by 60 s intervals.

**Days 2–4.** Extinction to an auditory CS/cage exposure. One rat from each pair (the partner) underwent extinction protocol (in context B), which consisted of initial 2 min habituation followed by exposure to 30 CS separated by 60 s intervals. The second rat from each pair (the subject) spent the same amount of time in the experimental cage (also in context B) but without a CS presentation.

**Day 5.** Test of fear extinction: Both rats, the fear unextinguished subject and its extinguished partner were placed in the experimental cage (separated by wire mesh as described above) and after 120 s of habituation were exposed to 15 CS (separated by 60 s intervals) in context B.

Several variants of this test paradigm were used:

- Alone—both rats, fear unextinguished subject and its extinguished partner were tested alone on day 5
- Conditioned together—on day 1, both animals: a subject and a partner were subjected to fear conditioning simultaneously (not alone as in the rest of the protocols) in adjacent chambers of the apparatus (where they could see, smell and hear each other throughout the entire conditioning session). The following protocol was as described above: the partner rat was fear extinguished during 3 subsequent sessions of extinction, while the subject rat was exposed to the experimental cage and on day 5 both rats were tested together.
- With an anesthetized partner—during the extinction test on day 5 the fear extinguished partner was anesthetized and placed in the middle of the experimental chamber adjacent to the chamber of the tested rat.
- No visual cues—rats were separated by a plastic opaque partition (instead of metal wire mesh used in other variants of the experiment) during the entire test; the partition blocked the visual cues but did not prevent olfactory communication as a gap below the partition allowed animals to smell their partners.
- Both rats not extinguished—in this variant, not only the subject but also its partner did not undergo extinction on days 2–4. Instead, they were exposed to the experimental cage and then tested together on day 5.
- With a naïve partner—a partner who was a cagemate of a fear unextinguished subject was not previously fear conditioned.
- With an unfamiliar, naïve partner—fear unextinguished subjects were tested not with their cagemates, but with another, unfamiliar male rat of the same age, sex, strain (Wistar), and colony, the partner rats were not fear conditioned.
- With a naïve partner of another strain—fear unextinguished subjects were tested with a male from a different rat strain (Long Evans); the partner rats were not fear conditioned.

### Analysis of behavioral data

Freezing was defined as at least 1 s of immobility and scored automatically in offline videos using BehaActive software (P. Boguszewski, Andrzejewski et al. 2020). We analyzed freezing in the CS + 20 s window because our pilot analyses of video recordings showed that it gives the least variable results, which stems from the fact that freezing that starts during the CS often lasts several seconds after CS offset. For more detailed analysis, behavior was manually scored by trained observers, with frame-to-frame temporal resolution using BehaView Software (P. Boguszewski, http://www.pmbogusz.net/?a=behaview). The ethogram included the following behaviors: quiescence—when rats stayed motionless, mesh sniffing—when rats sniffed the wire mesh divider, their partners were in some distance from the divider, prosocial behaviors—when rats directly contacted their partners via the mesh, cage exploration—when rats moved horizontally investigating non-social cues in the experimental cage, and rearing—when rats stood on hind legs exploring the cage. Ultrasound vocalizations (USV) recordings (for details, see Kondrakiewicz et al. 2019) were analyzed with RatRec software (Miron Kursa, Adam Hamed, Hamed et al. 2009).

### Recurrence analysis

The freezing data were down-sampled to 1 Hz. Then the probability of freezing co-occurrence in both animals was computed for different time lags (±30 s) using a MATLAB function developed by López Pérez et al. (2017). To control for the possibility of random freezing co-occurrence, the same computation was additionally repeated 100 times after shuffling the data points from the non-extinguished rat in time. The results were averaged to obtain one control profile for each tested pair. Finally, to test on the group level if the observed co-freezing probabilities were higher than in the shuffled control, the respective probabilities were compared using paired *t* tests (separately for each time point, followed by the false discovery rate correction – FDR). The profiles presented in Figure 3 represent mean probability of co-freezing (average from all pairs in a group) together with standard error of the mean.

### c-Fos immunohistochemistry

Ninety minutes after the first CS delivered on the day 5 of the extinction tests, rats were sacrificed with an overdose of Morbital (Biowet, Pulawy; 133.3 mg/ml sodium pentobarbital, 26.7 mg/ml pentobarbital, i.p. injection), and transcardially perfused with ice-cold 0.1 M PBS (pH 7.4, Sigma), followed by 4% (wt/vol) paraformaldehyde (POCh) in PBS (pH 7.4). The brains were removed and stored in the same fixative for 24 h at 4°C, and subsequently immersed in 30% (wt/vol) sucrose at 4°C. Next the brains were slowly frozen and sectioned at 40 μm on a cryostat. The coronal brain sections containing the prefrontal cortex and the amygdala were collected. Immunofluorescence staining for c-Fos was performed on selected free-floating sections. The sections were washed 3 times in PBS (pH 7.4), incubated for 10 min in 0.03% H_2_O_2_ in PBS, washed twice in PBS, and incubated with a polyclonal antibody (anti-c-Fos, 1:1,000; Santa Cruz Biotechnology no. sc-52), in PBS and normal goat serum (3%; Vector) for 48 h at 4°C. The sections were then washed 3 times in PBS with 0.3% Triton X-100 (Sigma), incubated with goat anti-rabbit biotinylated secondary antibody (1:500; Vector) in PBS/Triton and normal goat serum (3%) for 2 h at room temperature, washed 3 times in PBS/Triton, incubated with avidin–biotin complex (1:1,000 in PBS/Triton; Vector ABC kit) for 1 h at room temperature, and washed 3 times in PBS. The immunostaining reaction was developed using the oxidase-diaminobenzidine-nickel method. The sections were incubated in distilled water with diaminobenzidine (DAB; Sigma), 0.5 M nickel chloride, and peroxidase (Sigma) for 5 min. The staining reaction was stopped by three washes with PBS. The reaction resulted in a dark-brown stain within the nuclei of c-Fos immunoreactive neurons. The sections were mounted on slides, air dried, dehydrated in ethanol solutions and xylene, and cover slipped with Permount (Fisher Chemicals).

### Image capture and quantification of c-Fos expression

The measure of c-Fos immunopositivity was expressed as density, determined in the following manner: for each brain section, the number of c-Fos immunopositive nuclei in areas of interest was counted and divided by the area occupied by the structure (in millimeters squared). Image analysis was done with the aid of Nikon Eclipse Ni-U microscope and an image analysis computer program (ImageJ, NIH USA) on 2 sections per animal brain. The analysis was processed by experimenters who were blind to the treatments during image acquisition as well as during cell counting. Boundaries of each structure were defined with the use of the Paxinos atlas (Paxinos and Watson 2006). The borders of ACC (A24, anterior part), IL (A25) and PL (A32) were defined by the shape of the corpus callosum (van Heukelum et al. 2020), which served as a guideline for choosing the appropriate, corresponding atlas plate. For the LA, BA, CeAm, and CeAl, the shape of the amygdala itself was used as a guideline.

### Surgical procedures

#### Virus injections and optic fibers implantations

The adeno-associated viral vector used rAAV5/CamKIIa-eNpHR3.0-eYFP was purchased from Virus Vector Core. Viral titers were 4.9 × 10^12^ particles/ml. Fourteen days before the behavioral training, the experimental and control rats received bilateral intracranial injections of the virus [experimental: rAAV5/CamKIIa-eNpHR3.0-eYFP, control: rAAV5/CamKIIa-eYFP] into the IL. All surgical instruments were sterilized before surgery. Rats were anesthetized with isoflurane (Aerane, 5% induction, 1% for maintenance), subcutaneously injected with an analgesic (Butorfanol, 1 mg/kg) and placed into the stereotaxic apparatus (David Kopf Instruments). Eyes were moistened with ocular lubricant and the scalp was shaved. Next the scalp was disinfected with 70% (vol/vol) alcohol, incised, and skin was retracted. Two small burr holes were drilled to allow for a 1 μL NanoFil syringe needle (World Precision Instruments) to be lowered into the desired part of the brain.

The coordinates used for drilling 2 small burr holes were as follows: IL [anteroposterior (AP): +3.2, mediolateral (ML): ±0.6, dorsoventral (DV): −2.2]. The needles were placed at an angle of 30°. The virus was delivered using a Nanofil syringe and infusion pump (MicroSyringe Pump; World Precision Instruments; volume 500 nl; speed 100 nl/min; for 5 min; the needle remained in place for another 5 min to allow for the virus diffusion). Additional 3 small holes were drilled and 3 skull screws were placed to secure the cement cap. After the viral injection, optic fiber cannulas were mounted (ThorLabs fibers 0.39 NA, 200 μm core, mod. FT200UMT; placed in Ceramic Ferrules mod. CFLC230-10; connected to self-made threaded metal covers with Data Optics Hysol epoxy glue no. 0151). Optic fibers were inserted bilaterally into the IL at an angle of 30°. Then, the exposed skull was coated with dental cement. All the animals were administered an analgesic/anti-inflammatory (Tolfedine; 4 mg/kg; s.c.) and antibiotic (Baytril; 2.5 mg/kg; s.c.). To avoid dehydration, the animals were given 1 mL of warm 0.9% NaCl/100 g of body weight by s.c. injection. The rats were kept on a heating pad until they recovered from anesthesia before returning to their home cages.

#### Optogenetic experiment

The optogenetic inhibition was done on day 5 when animals were tested together. The unextinguished subject rat implanted with optic fibers was subjected to photoinhibition during all 15 CS (and 2 times after the last CS). The laser-on phases began 20 s prior to the CS presentation and ended after the cessation of CS presentation (40 s total). Laser illumination was initiated 20 s before tone because long-response latencies in some IL neurons following laser illumination had been previously observed (Do-Monte et al. 2015). In the control group, subject rats injected with a control virus (not encoding any opsins) were treated in the same way.

A yellow laser with a wavelength of 589 nm and maximum output of 100 mW (CNI) was used. The laser light was delivered continuously through a rotary joint (Doric Lenses) to two optic fibers (Thorlabs—core 200 μm, NA 0.37, 2 m in length) and from there to thread-attached self-made optic fiber cannulas. The typical laser power at the cannula tip was 10 mW. The laser onset was triggered by a transistor-transistor logic (TTL) output from the conditioning chamber (Med Associates). The laser was controlled by an Arduino board. The laser power output was measured using a commercial power meter (Thorlabs model PM200 with S121C sensor).

#### Histological verification of location of optic fiber and vector injections

Animals were deeply anesthetized with an overdose of sodium pentobarbital (100 mg/kg) and perfused transcardially with 0.01 M PBS (200 mL) and 4% PFA (paraformaldehyde in PBS, 300 mL). Brains were stored for 24 h in PFA, then for 3 days in 30% sucrose and sectioned with a freezing microtome into 40 μm slices. Yellow fluorescent protein (YFP) expression in injection sites and fiber optic placement was checked for each animal.

#### Chemogenetic inhibition

For chemogenetic experiments 2 stereotactic (Kopf Instruments) surgeries have been performed with the 2–3 weeks break between them. First surgery consisted of bilateral injection of 500 nl per hemisphere of AAV-hSyn-DIO {hCAR}off-{hM4Di-mCherry}on-W3SL (Addgene plasmid # 111397) into vHIP at AP: –5.3, ML: ±5.5, DV: −7 and −6.8 (250 nl at each depth level) or ACC: AP: 1.2, ML: ±0.8, DV: −1.8. Virus was infused at a rate of 100 nl/min with the needle left at the injection site for 5 min after each infusion. Second surgery consisted of bilateral injections of CAV2-Cre-GFP or CAV2-Cre without fluorescent protein (Plateforme de Vectorologie de Montpellier, 300 nl per hemisphere) into PL at AP: 2.8, ML: ±1.55 and DV: −2.7 (at an 20° angle) or into CeA at: AP: –1.9, ML: ±3.95, DV: −7.8 at a rate of 100 nl/min with needle left at injection site for 5 min after infusion. All injections have been done using Nanofil syringe (World Precision Instruments) with NF33BV-2 needle. After 1 week of recovery, rats underwent habituation and the behavioral procedure as described above.

On the 5th day of the behavioral experiment (14–16 days after second surgery) subjects (fear unextinguished rats) were injected intraperitoneally with C21 compound (3 mg/kg, the concentration that does not have off target effects (Jendryka et al. 2019), diluted in NaCl) 30 min prior to experiment. In control groups, subject rats got the same amount of NaCl solution. Histological verification of vector injections was performed as in the optogenetic experiment.

#### Open field

To assess the potential effects of C21 on locomotor activity and anxiety, we tested the animals in an open field. The rat was placed in the center of the 1 m x 1 m x 40 cm gray dimly lit (25lux) arena and allowed free exploration for 7 min. After testing of each rat, the arena was cleaned with 70% ethanol. C21 or NaCl was injected 30 min before the onset of the experiment. The behavior was recorded with a FLIR BFS-U3-16S2M-CS camera mounted above the arena and analyzed offline with DeepLabCut and a python script. We analyzed velocity and distance covered in the open field, the number of entries and total time spent in the center of the open field.

### Statistical analysis

For statistical analyses of raw data GraphPad Prism6 and Matlab software with Statistical package and custom-made scripts were used, and *P* < 0.05 was considered significant. Data were presented as mean ± SEM. Freezing to CS was calculated within CS and subsequent 20 s and transformed into the percentage values. Normality of distributions was analyzed with Shapiro–Wilk test. Comparisons of freezing level between 2 last CS of fear conditioning and extinction test were done using paired *t* test or Wilcoxon matched-pairs signed rank test in case of lack of normality of distribution. A comparison of freezing levels during the pre-CS period between rats tested with partners and rats tested alone was made using the Mann–Whitney test. For comparisons of all groups in behavioral tests, as the assumption of normality was not met by all datasets, the average freezing levels during 2 last CS of fear conditioning, averaged freezing levels to CS during fear extinction, quiescence, mesh sniffing, prosocial behavior, cage exploration, and rearing analyzed throughout the whole session, as well as numbers of 22 kHz and 50 kHz USVs were analyzed with permuted Analysis of Variance (ANOVA) followed by permuted *t* test (Delorme and Makeig 2004). Note that for the permutation tests, the *F* and *t* statistics and the degrees of freedom are provided for reference only as the permutation procedure estimated the *P* values. Responses to subsequent CS were analyzed with mixed repeated-measures ANOVA, followed by planned comparisons with the reference groups. All planned comparisons were corrected for multiple comparison using FDR (Benjamini and Yekutieli 2001). c-Fos expression was analyzed with repeated-measures ANOVA followed by FDR test. Opto- and chemo-genetic experiments: The photoinhibition dataset was analyzed with *t* test (average freezing level to the CS), and 2-way ANOVA (freezing in response to the subsequent CS). In the chemogentic experiments, we compared more than 2 groups simultaneously, so the same analysis were used as for behavioral experiments: freezing in response to the subsequent CS was analyzed with mixed repeated measures ANOVA and the rest of the results were analyzed with permuted ANOVA followed by permuted *t* test.

## Results

### Social buffering depends on its social component and does not equal contextual modulation of fear extinction

**Fig. 1:**
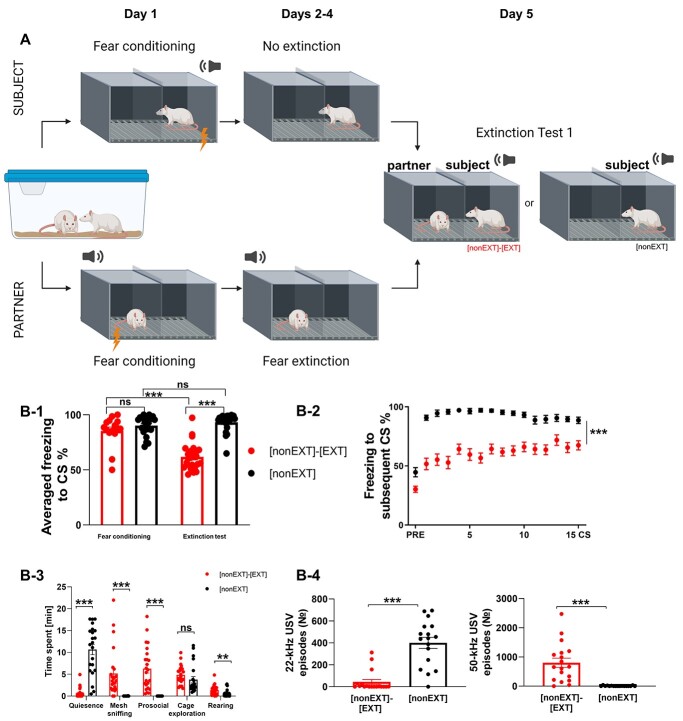
Presence of a partner strongly inhibits fear responses during fear extinction. (A) The experimental scheme. The rats were subjected to fear extinction test with a partner or separately. (B-1) Averaged freezing to CS, (B-2) freezing to subsequent CS. The freezing level during fear extinction test was lower in rats tested with a partner ([nonEXT]-[EXT], *n* = 24) than in rats tested separately ([nonEXT], *n* = 23). The dots on the freezing graphs (B-1) represent the mean freezing level to all CS presented for each animal. (B-3) Rats tested with a partner engaged in significantly more mesh sniffing, prosocial, and rearing (*n* = 24), while rats tested alone were mainly motionless (*n* = 23). (B-4) The animals tested in pairs (*n* = 18) produced more positive, 50-kHz USVs but less negative, 22-kHz USVs than those tested alone (*n* = 17). The graphs show means ± SEM, ^*^^*^P < 0.01, ^*^^*^^*^P < 0.001. For the description of the experimental groups, please refer to Table S1.

**Fig. 2:**
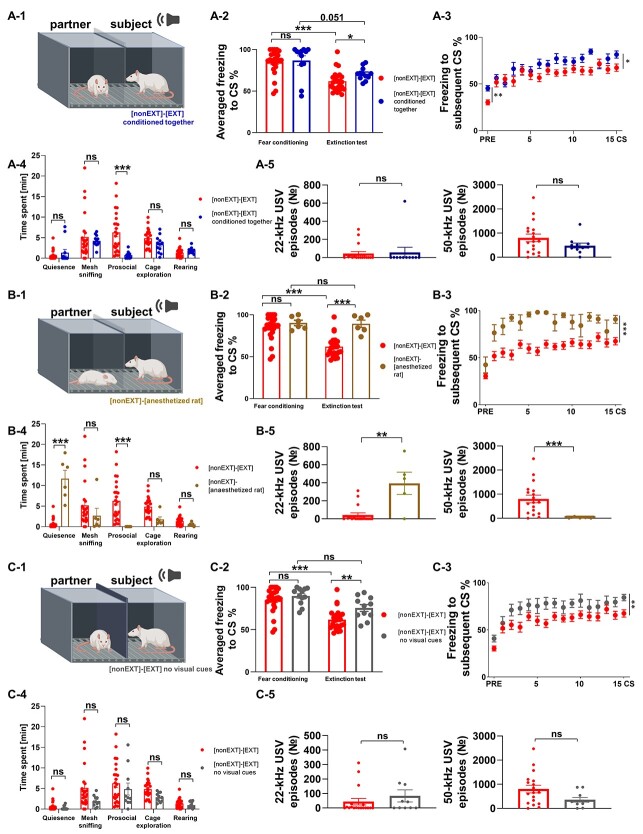
The magnitude of social buffering of fear by different partners and in different conditions. (A-1) The scheme of the extinction test [nonEXT]-[EXT]—conditioned together. (A-2) Averaged freezing to CS, (A-3) freezing to subsequent CS. The freezing level was higher in rats trained and tested in the same social context ([nonEXT]-[EXT]—conditioned together, *n* = 12) than in animals, in which the social context changed between the training and testing ([nonEXT]-[EXT], *n* = 24, the same data as presented in Fig. 1, here shown to enable direct comparison). (A-4) Rats conditioned together with their partners exhibit less prosocial behavior. (A-5) The number of 22-kHz and 50-kHz USVs did not differ between the groups ([nonEXT]-[EXT]—conditioned together, *n* = 11; ([nonEXT]-[EXT], *n* = 18). (B-1) The scheme of the extinction test ([nonEXT]-[anesthetized rat]). (B-2) Averaged freezing to CS, (B-3) Freezing to subsequent CS. Rats tested with an anesthetized partner ([nonEXT]-[anesthetized rat], *n* = 6) showed much higher levels of freezing than rats tested with a non-anesthetized partner ([nonEXT]-[EXT]). (B-4) In rats tested with anesthetized partner, we observed almost no prosocial behavior in comparison with rats tested with moving partners. (B-5) Rats tested with an anesthetized partner (*n* = 5) emitted more 22-kHz USV calls and less 50-kHZ calls than rats tested with non-anesthetized partner ([nonEXT]-[EXT], *n* = 18). (C-1) The scheme of the extinction test [nonEXT]-[EXT]-no visual cues. (C-2) Averaged freezing to CS, (C-3) freezing to subsequent CS. Rats tested with a partner placed behind an opaque partition showed higher freezing levels ([nonEXT]-[EXT]—no visual cues, *n* = 11) than rats separated by wire mesh ([nonEXT]-[EXT]). (C-4) Detailed analysis of behavior revealed no differences between rats visually separated from their partners and rats separated by wire mesh. (C-5) Testing rats with an opaque partition did not change the number of 22-kHz or 55-kHz USVs ([nonEXT]-[EXT]-no visual cues, *n* = 10; [nonEXT]-[EXT], *n* = 18). The graphs show means ± SEM, ^*^P < 0.05, ^*^^*^P < 0.01, ^*^^*^^*^P < 0.001. For the description of the experimental groups, please refer to Table S1.

To test the role of social support in fear extinction, we subjected the rats to fear extinction either alone or with a partner. Before the fear extinction session, both rats were separately fear conditioned to tones. Then the partner rat underwent fear extinction in such a way that his freezing level during the last fear extinction session was low, whereas the subject rat was only exposed to the experimental cage without the CS presentation, i.e. it was not subjected to fear extinction (Fig. 1A). We first compared averaged freezing during last 2 CS of conditioning session between the groups (permuted ANOVA, *F*(8, 116) = 1.6446, *P* = 0.2167; for the detailed description of the groups, please refer to Table S1). Next, we did planned comparison between rats tested with a partner and tested alone. Freezing at the end of the fear conditioning did not differ between the groups (Fig. 1B-1, permuted *t* test, *t* = 1.7147, df = 43, not significant after FDR correction). The level of freezing during extinction test was lower than during two last CS in conditioning session only in subject rats tested in the presence of extinguished partners (Fig. 1B-1, Wilcoxon matched-pairs signed rank test, *W* = 232, *P* = 0.0004; rats tested alone: *W* = 100, *P* = 0.1341). Next, we compared freezing to CS, other behavioral measures and USVs between all the groups tested with different partners (cf. Figs. 1,2,4), and proceeded with planned comparisons between the groups. During the extinction test, there was a strong effect of group on the average freezing level to the CS [permuted ANOVA *F*(8, 118) = 10.78, *P* < 0.0001], as well as in freezing in response to the subsequent CS [mixed ANOVA, group: *F*(8, 1905) = 11.299, *P* < 0.0001, time: *F*(15,1905) = 35.4215, *P* < 0.0001, group x time: *F*(120,1905) = 1.9124, *P* < 0.0001]. Next, we compared other behavioral measures (see Methods) between all groups: quiescence [permuted ANOVA, *F*(8, 126) = 30.1460, *P* < 0.0001], cage exploration [permuted ANOVA, *F*(8, 126) = 2.7494, *P* = 0.0515], rearing [permuted ANOVA, *F*(8, 126) = 3.8452, *P* = 0.0058], mesh sniffing [permuted ANOVA, *F*(8, 126) = 7.3269, *P* = 0.0008], and prosocial behaviors [permuted ANOVA, *F*(8, 126) = 7.9415, *P* = 0.0003]. Finally, we compared the number of 22-kHz USVs [permuted ANOVA, *F*(8, 100) = 7.7865, *P* = 0.0002] and 50-kHz USVs [permuted ANOVA, *F*(8, 100) = 4.3197, *P* = 0.0051] between all groups.

**Fig. 3:**
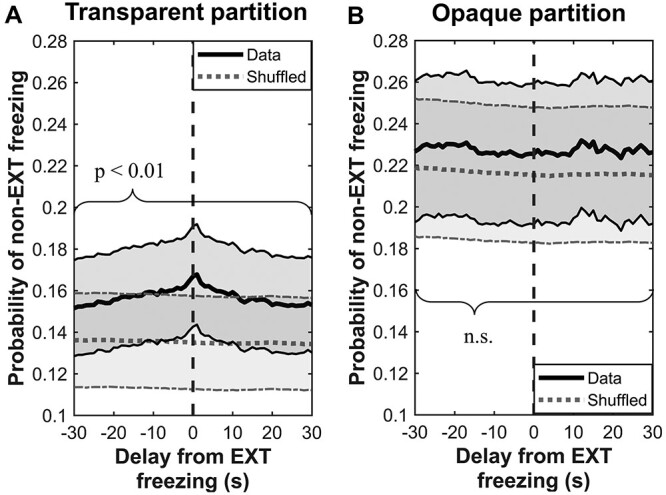
Freezing is synchronized between partners, but only when visual cues are available. (A) When tested in a cage with transparent partition, the non-extinguished subject rats had the highest chance of freezing if their extinguished partners were also freezing at a similar time. Moreover, the co-occurrence of both rats freezing was higher than expected at chance level (all *P* < 0.01, FDR adjusted) during the whole tested time window (±30 s). (B) No such relationship was observed when rats were separated with an opaque partition (n.s.). Thick red lines: average probability of freezing co-occurrence for a given time lag (±30 s); thick gray lines: similar probability calculated after shuffling data points from respective extinguished partners randomly across time; shaded red/gray areas: SEM calculated for either observed or shuffled data, respectively.

Next, we performed planned comparisons. Rats tested with a fear extinguished partner displayed much less freezing than rats tested separately (Fig. 1B-1, permuted *t* test, *t* = 9.7736, df = 43, *P* < 0.0001). The effect was also significant in response to subsequent CS [Fig. 1B-2; mixed ANOVA, group: *F*(1, 675) = 105.5065, *P* < 0.0001, time: *F*(15, 675) = 24.9186, *P* < 0.0000, group x time: *F*(15, 675) = 4.0985, *P* < 0.0001]. Detailed behavioral analysis showed that the animals tested in pairs spent more time on social exploration (Fig. 1B-3, prosocial, i.e. direct contact with a partner through the mesh: permuted *t* test, *t* = 6.0175, df = 44, *P* < 0.0001; mesh sniffing: permuted *t* test, *t* = 4.4223, df = 44, *P* < 0.0001), and non-social exploratory behavior (rearing: permuted *t* test, *t* = 2.7, df = 44, *P* = 0.0085), whereas those tested alone spent more time motionless (quiescence: permuted *t* test, *t* = 8.702, df = 44, *P* < 0.0001). For the statistical analysis of other behavioral parameters, see Table S2. In the animals tested with a partner, visual inspection of video recordings of extinction test revealed that subject rats decreased freezing level mostly at the beginning of extinction test session due to interaction with partners. Freezing appears to be increasing within the session as a function of decreased social interaction and increased freezing in response to the CS (see Fig. 1B-2).

**Fig. 4:**
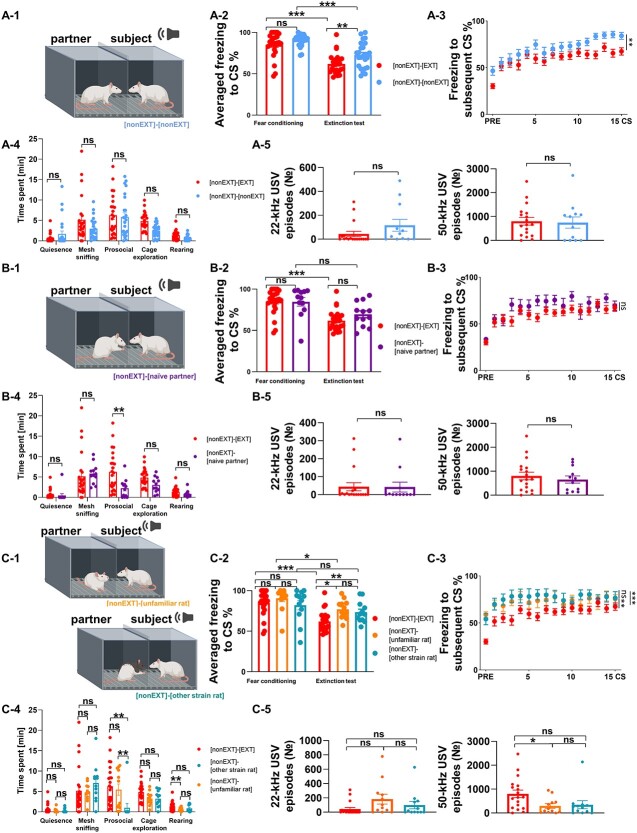
The magnitude of social buffering of fear depends on the emotional status of a partner rat, familiarity, and strain identity of animals. (A-1) The scheme of the extinction test [nonEXT]-[nonEXT]. (A-2) Averaged freezing to CS, (A-3) freezing to subsequent CS. Rats tested with partners subjected earlier to fear extinction ([nonEXT]-[EXT], *n* = 24, the same data as presented in Fig. 1, here shown to enable direct comparison) had lower freezing levels than the animals paired with partners not subjected to fear extinction ([nonEXT]-[nonEXT], *n* = 24). (A-4) Rats tested in the presence of fear unextinguished partners spent similar amount of time on each behavior. (A-5) The number of 22-kHz and 50-kHz USVs did not differ between rats tested with partners subjected to fear extinction (*n* = 24) and tested with partners not subjected to fear extinction (*n* = 12). (B1) The scheme of the extinction test [nonEXT]-[naïve partner]. (B-2) Averaged freezing to CS, (B-3) freezing to subsequent CS. The freezing level was similar in rats paired with partners subjected to three extinction sessions ([nonEXT]-[EXT]) and naive partner ([nonEXT]-[naïve partner], *n* = 12). (B-4) Rats tested with a partner previously subjected to fear extinction spent more time on prosocial behaviors than rats tested with a naïve partner. (B-5) The number of 22-kHz and 50-kHz USVs did not differ between the groups ([nonEXT]-[EXT], *n* = 18 and [nonEXT]-[naïve partner], *n* = 12). (C-1) The scheme of the extinction test ([nonEXT]-[other strain rat] and [nonEXT]-[unfamiliar rat]. (C-2) Averaged freezing to CS, (C-3) freezing to subsequent CS. Rats tested with a familiar partner ([nonEXT]-[EXT]) showed lower freezing than either the animals paired with unfamiliar partners ([nonEXT]-[unfamiliar rat], *n* = 12) of the same strain or with unfamiliar partners of a different strain ([nonEXT]-[other strain rat], *n* = 12). (C-4) Detailed analysis of behavioral data revealed less rearing behavior in rats tested with an unfamiliar partner than in animals tested with a familiar partner. Rats tested with a partner of a different strain presented less prosocial behavior than rats tested with a familiar or an unfamiliar partner of the same strain. (C-5) The number of 50 kHz USV calls was lower in rats tested with unfamiliar partners (*n* = 12) of the same strain, but not of a different strain (*n* = 12) in comparison with rats tested with their cagemate (*n* = 18). The dots on the freezing graphs represent the mean freezing level during the whole testing session for each animal. The graphs show means ± SEM, ^*^P < 0.05, ^*^^*^P < 0.01, ^*^^*^^*^P < 0.001. For the description of the experimental groups, please refer to Table S1.

Further, we recorded the number of ultrasonic vocalizations (USVs) at 2 frequencies, around 22-kHz and around 50-kHz, emitted during test sessions. As the USVs at the frequency of around 22 kHz appear in highly stressful situations (e.g. during fear conditioning) and high-frequency vocalizations are associated with more positive emotional valence (Brudzynski 2013; Simola and Granon 2019) we treated USVs as an additional measure of the emotional state of tested animals. We observed that the rats accompanied by a partner produced much more high-frequency (positive) USVs than the animals tested alone (Fig. 1B-4, permuted *t* test, *t* = 4.8986, df = 33, *P* < 0.0001), and less low-frequency (negative) USVs (Fig. 1B-4, permuted *t* test, *t* = 6.6120, df = 33, *P* < 0.0001).

**Fig. 5:**
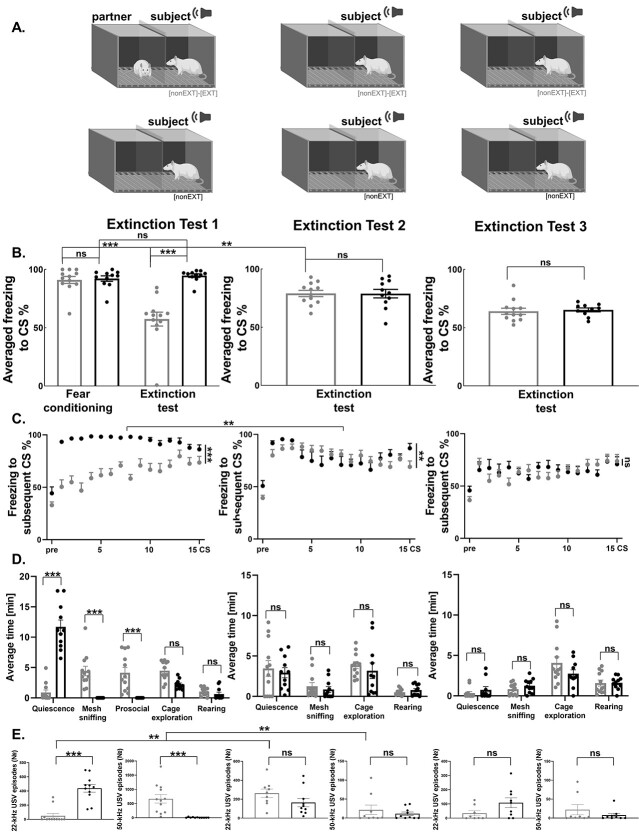
Social buffering does not improve fear extinction memory. (A) The scheme of the extinction test. (B) The level of freezing was lower during the accompanied ([nonEXT]-[EXT], *n* = 12) than the solitary ([nonEXT], *n* = 11) extinction session (extinction test 1). However, when the rats were tested alone on 2 consecutive days (extinction test 2, [nonEXT]-[EXT], *n* = 12, extinction test 3, [nonEXT]-[EXT], *n* = 12), the average freezing level to the CS in the groups with the accompanied and the solitary extinction (extinction test 2, [nonEXT], *n* = 11, extinction test 3, [nonEXT], *n* = 11) did not differ. The dots represent the mean freezing level to the CS for each animal. (C) Freezing levels to the subsequent CS. (D) The detailed analysis of behavior of the rats subjected to accompanied and unaccompanied extinction session. During extinction test 1 mesh sniffing and prosocial behavior were significantly higher in rats tested with a partner than during the solitary extinction session, while quiescence was significantly lower. No differences were detected during extinction tests 2 and 3. (E) Rats subjected to fear extinction with a companion (extinction test 1, [nonEXT]-[EXT], *n* = 12) emitted less 22-kHz and more 55-kHz USVs than the animals tested alone (extinction test 1, [nonEXT], *n* = 11). No differences were found during extinction tests 2 and 3. The graphs show means ± SEM, ^*^^*^P < 0.01, ^*^^*^^*^P < 0.001.

Physical context is a critical modulator of fear extinction (Bouton et al. 2006). The conditioned freezing response tends to be lower when tested in a physical context different from that in which conditioning took place. In the experiments carried out on single animals, the physical context is usually changed by manipulating scent, lighting, and background noise to distinguish the conditioning and testing chamber (Chang et al. 2009). In our experiments we applied such a standard protocol including physical context change. However, introducing the partner rat to the adjacent chamber during the extinction test session can also add to the physical changes of the context, which could lower freezing. Thus, to investigate whether the diminished fear response we observed was caused by this additional change in the context, we kept the conditioning and testing social context the same by training and testing the subject with the partner rat. To test this, we subjected rats to fear conditioning in pairs rather than alone, which meant having the same social context during training and extinction test sessions (Fig. 2A-1, the rest of the procedure was the same as above). We hypothesized that if the inhibition of fear we observed depends on the context change, we should see higher freezing during the extinction session in rats trained and tested in the same social context because the conditioning and extinction contexts were more similar to each other. The rats conditioned together showed the same level of freezing at the end of the session as the rats conditioned separately (Fig. 2A-2, the freezing averaged across the last two CS, permuted *t* test, *t* = 0.6736, df = 32, not significant after FDR correction). The level of freezing of the rats conditioned together tended to be lower during the extinction test than during 2 last CS of conditioning session (Fig. 2A-2, Wilcoxon matched-pairs signed rank test, *W* = 50, *P* = 0.0508). During the extinction test the rats conditioned together had higher average freezing level to the CS than rats conditioned separately (Fig. 2A-2, permuted *t* test, *t* = 1.8697, df = 32, *P* = 0.0207), and higher freezing in response to the subsequent CS [Fig. 2A-3, mixed ANOVA, group: *F*(1, 510) = 6.2226, *P* = 0.0175, time: *F*(15,510) = 10.4845, *P* < 0.0001]. In line, the rats conditioned together with their partners (with no context change between the conditioning and extinction sessions) showed higher freezing to experimental context than the rats conditioned separately (freezing measured during the 2-min pre-CS period, Fig. 2A-3, Mann-Whitney test, *U* = 50, *P* = 0.0010). Although we observed increased freezing in rats trained and tested in the same social context, the freezing level in these conditions was still significantly lower than in single-tested animals (Figs. 1B-2 and 2A-2, average freezing level to the CS, permuted *t* test *t* = 8.1398, df = 31, *P* < 0.0001, Figs. 1B-3 and 2A-3, freezing to the subsequent CS, mixed ANOVA, group: *F*(1, 495) = 57.6265, *P* < 0.0001, time: *F*(15,510) = 25.5423, *P* < 0.0001, group x time: *F*(15, 495) = 9.1396, *P* < 0.0001). The detailed analysis of behavior showed that rats conditioned together display less prosocial behavior, i.e. direct contact with their partners via the mesh (Fig. 2A-4, permuted *t* test, *t* = 6.0175, df = 44, *P* < 0.0001); for other analyses see Table S2. We did not observe any difference in the number of either 22-kHz USVs (Fig. 2A-5, permuted *t* test, *t* = 0.2513, df = 27, not significant after FDR correction), or 50-kHz (Fig. 2A-5, permuted *t* test, *t* = 1.465, df = 27, not significant after FDR correction) between the groups. Thus, we observed a smaller decrease in freezing in rats trained and tested together than in rats trained separately, suggesting some role of physical context change in the observed effect. However, the freezing level was still significantly lower than in rats tested alone. Thus, these results indicate that the change of the context is not a sufficient explanation of the social effects we observed, and that social buffering depends on social rather than just contextual factors. To test this hypothesis further, we investigated the level of fear during extinction in animals paired with an anesthetized rat or with a rat placed behind an opaque partition.

In the next experiment, the partner of fear unextinguished subject was anesthetized and placed in the middle of his part of experimental chamber. The level of freezing during the extinction test was significantly higher in rats tested with an anesthetized partner compared to rats tested with a moving partner (Fig. 2B-1). Freezing at the end of the fear conditioning session did not differ between the groups (Fig. 2B-2, freezing averaged across the last two CS, permuted *t* test, *t* = 0.5064, df = 26, not significant after FDR correction). The level of freezing in the subject tested with an anesthetized partner was not lower during the extinction test than during 2 last CS of conditioning session (Fig. 2B-2, paired *t* test, *t* = 0.3385, df = 5, *P* = 0.7487). During the extinction test the rats tested with an anesthetized partner had higher freezing compared to rats tested with a moving partner [Fig. 2B-2, average freezing level to the CS, permuted *t* test, *t* = 4.1814, df = 26, *P* = 0.0005; Fig. 2B-3, freezing in response to the subsequent CS, mixed ANOVA, group: *F*(1, 420) = 24.5284, *P* < 0.0001, time *F*(15, 420) = 7.6098, *P* < 0.0001]. The presence of an anesthetized partner rat (Fig. 2B-2, B-3) did not reduce the freezing level compared to rats tested separately [Fig. 1B-2, B-3; average freezing level to the CS, permuted *t* test, *t* = 1.3837, df = 25, not significant after FDR correction; freezing in response to the subsequent CS, mixed ANOVA, group: *F*(1, 405) = 0.9987, not significant after FDR correction, time: *F*(15, 405) = 30.4179, *P* < 0.0001, group x time: *F*(15, 405) = 1.5184, not significant after FDR correction]. Detailed analysis of behavioral data confirmed that the animals tested with a non-anesthetized partner spent more time on prosocial behavior (Fig. 2B-4, permuted *t* test, *t* = 3.1017, df = 28, *P* < 0.0001), whereas those tested in the presence of an anesthetized partner spent more time motionless (quiescence: permuted *t* test, *t* = 10.4081, df = 28, *P* < 0.0001). Further, unextinguished subjects in the presence of an anesthetized rat produced more 22-kHz USVs (Fig. 2B-5, permuted *t* test, *t* = 4.6782, df = 21, *P* = 0.0031), and less 50-kHz (Fig. 2B-5, permuted *t* test, *t* = 2.5699, df = 21, *P* = 0.0009) than rats tested with a moving partner.

Elimination of the visual cues from the partner rat by placing a non-transparent separator (perception of acoustic and olfactory cues was still possible through a gap below the partition and around the doors in the front of experimental cage, Fig. 2C-1) resulted in a reduced social buffering effect. The level of freezing at the end of fear conditioning session was similar as in the rats tested with a wire mesh partition (Fig. 2C-2, the freezing averaged across the last 2 CS, permuted *t* test, *t* = 0.0884, df = 31, not significant after FDR correction). However, in rats tested with partners separated by opaque partition the level of freezing did not decrease during extinction test comparing to the freezing level during the last 2 CS of fear conditioning (Fig. 2C-2, Wilcoxon matched-pairs signed rank test, *W* = 20, *P* = 0.4131). During the extinction test, rats tested with a non-transparent separator had a higher freezing level than rats tested with a partner behind wire mesh partition [Fig. 2C-2, average freezing level to the CS, permuted *t* test, *t* = 3.2271, df = 31, *P* = 0.0015; Fig. 2C-3, freezing in response to the subsequent CS, mixed ANOVA, group: *F*(1, 495) = 9.3991, *P* = 0.0044, time: (F1, 15) = 9.4167, *P* < 0.0001]. Number of 22-kHz and 50-kHz USV calls did not differ between the groups [Fig. 2C-5, number of 22-kHz USVs: permuted *t* test, *t* = 0.9207, df = 26, not significant after FDR correction; number of 50-kHz USVs: permuted *t* test, *t* = 1.9770, df = 26, not significant after FDR correction]. For the results of other statistical comparisons, please refer to Table S2.

Additionally, reducing visual input changed the distribution of freezing episodes across time. The correlation between fear responses of rats that were not subjected to extinction and their fear extinguished partners depended on the availability of visual cues: it was strong when animals could see each other through a transparent partition and low when animals were speared by an opaque divider (Fig. 3).The results confirmed that the presence of a freely behaving partner is crucial for the inhibition of fear during extinction and that visual stimuli partially mediate the observed effect.

### The magnitude of social inhibition of fear during fear extinction depends on the emotional status of a partner rat, familiarity, and strain sameness of animals

To test how different social factors modulate the magnitude of social buffering during fear extinction, we manipulated the partner rats’ emotional status, familiarity, and strain identity. The extent of the social buffering effect depended on the emotional status of the partner rat. To test for the role of the partner rats’ emotional status, the partner rats, unlike in other experiments, were not subjected to fear extinction before the extinction test (Fig. 4A-1). We compared the groups of subjects tested with partners either subjected to three extinction sessions before or not subjected to fear extinction. The level of freezing at the end of fear conditioning session was similar in both groups (Fig. 4A-2, the freezing averaged across the last 2 CS, permuted *t* test, *t* = 2.2337, df = 42, not significant after FDR correction). In the group in which both subject and partner were unextinguished, the average level of freezing during the extinction test was significantly lower than during the two last CS of conditioning (Fig. 4A-1, Wilcoxon matched-pairs signed rank test, *W* = 219, *P* < 0.0001). However, the partners previously subjected to 3 extinction sessions were more effective social buffers than the rats not subjected to fear extinction; the subjects paired with them showed less freezing [Fig. 4A-2, average freezing level to the CS, permuted *t* test, *t* = 2.7792, df = 44, *P* = 0.0075; Fig. 4A-3, freezing in response to the subsequent CS, mixed ANOVA, group: F(1, 690) = 8.0216, *P* < 0.0069, time: *F*(15,690) = 15,0266, *P* < 0.0001]. For the results of other statistical comparisons, please refer to Table S2. In contrast, there were no significant differences in the number of 22-kHz and 50 kHz USVs [Fig. 4A-5, number of 22-kHz USVs: permuted *t* test, *t* = 1.4951, df = 28, not significant after FDR correction; number of 50-kHz USVs: permuted *t* test, *t* = 0.1924, df = 28, not significant after FDR correction]. Next, we compared the level of freezing in rats paired during the extinction test with partners previously subjected to 3 extinction sessions and naïve cagemates (not subjected to fear conditioning before, Fig. 4B-1). The level of freezing at the end of fear conditioning session was similar in both groups (Fig. 4B-2, the freezing averaged across the last 2 CS, permuted *t* test, *t* = 0.5786, df = 32, not significant after FDR correction). The level of freezing during the extinction test did not significantly diminish in rats tested with naïve partner (Fig. 4B-2, the freezing averaged across the last 2 CS compared with averaged freezing during extinction test, Wilcoxon matched-pairs signed rank test, *W* = 36, *P* = 0.1763). However, the freezing level of rats tested with naïve partners did not differ in comparison with animals tested with previously experienced partners [Fig. 4B-2, average freezing level to the CS, permuted *t* test, *t* = 1.2104, df = 32, not significant after FDR correction; Fig. 4B-3, freezing in response to the subsequent CS, mixed ANOVA yielded a non-significant effect of group (after FDR correction), a significant effect of time *F*(15,510) = 5.4381, *P* < 0.0001, and no significant interaction of the 2 F(15,510) = 0.9200, not significant after FDR correction]. The presence of a partner previously subjected to fear extinction resulted in more prosocial behaviors [direct contact with a partner through the mesh, Fig. 4B-4, permuted *t* test, *t* = 2.6925, df = 34, *P* = 0.0067]. For other statistical comparisons, see Table S2. Finally, there were no differences in the number of 22-kHz and 50-kHz USVs between rats tested with a partner with 3 fear extinction sessions and naive ones (Fig. 4B-5, number of 22-kHz USVs, permuted *t* test, *t* = 0.0470, df = 28, not significant after FDR correction; number of 50-kHz USVs, permuted *t* test, *t* = 0.6463, df = 28, not significant after FDR correction). Thus, the partners subjected to fear extinction and naïve ones were similarly effective as social buffers.

Familiarity and the strain belonging also modulated the social buffering effect (Fig. 4C-1). We compared familiar and unfamiliar rats belonging to the same strain, and unfamiliar rats belonging to the same and to different strain. The level of freezing at the end of fear conditioning session was similar in rats tested with familiar partner and tested with unfamiliar partner belonging to the same strain (Fig. 4C-2, the freezing averaged across the last 2 CS, permuted *t* test, *t* = 1.0782, df = 32, not significant after FDR correction), and with partner from different strain (Fig. 4C-2, the freezing averaged across the last 2 CS, permuted *t* test, *t* = 0.8186, df = 32, not significant after FDR correction). The freezing level during fear conditioning also did not differ between rats tested with unfamiliar partners from the same strain and with unfamiliar partners from different strain (Fig. 4C-2, the freezing averaged across the last 2 CS, permuted *t* test, *t* = 1.5220, df = 20, not significant after FDR correction). During the extinction session the freezing level diminished in group where subjects were tested with unfamiliar partners of the same strain (Fig. 4C-2, the freezing averaged across the last 2 CS compared with averaged freezing during extinction test, Wilcoxon matched-pairs signed rank test, *W* = 60, *P* = 0.0161), but not in group where partner was unfamiliar and from different strain (Fig. 4C-2, Wilcoxon matched-pairs signed rank test, *W* = 6, *P* = 0.8501). The familiar partners were more effective buffers than unfamiliar animals during fear extinction, regardless of the partner’s strain; the freezing level was higher in rats tested with an unknown partner of the same strain (Fig. 4C-2, average freezing level to the CS, permuted *t* test, *t* = 3.3693, df = 32, *P* < 0.0019; Fig. 4C-3, freezing in response to the subsequent CS, mixed ANOVA, group: *F*(1, 510) = 14.6923, *P* < 0.0006, time: *F*(15,510) = 5.4381, *P* < 0.0001), and with an unknown partner of a different strain (Fig. 4C-2, average freezing level to the CS, permuted *t* test, *t* = 2.4551, df = 32, *P* = 0.0177; Fig. 4C-3, freezing in response to the subsequent CS, mixed ANOVA, group: *F*(1, 510) = 8.9705, *P* < 0.0006, time: *F*(15,510) = 5.1183, *P* < 0.0001). In contrast, there was no difference in freezing level between rats tested with unfamiliar partners of the same or a different strain (Fig. 4C-2, average freezing level to the CS, permuted *t* test, *t* = 0.6686, df = 25, not significant after FDR correction; Fig. 4C-3, freezing in response to the subsequent CS, mixed ANOVA, group: *F*(1, 330) = 0.4144, not significant after FDR correction, time: *F*(15, 330) = 2.2508, *P* = 0.0051, group x time: *F*(15, 330) = 0.3395, not significant after FDR correction]. More detailed analysis of the behavioral data revealed that rats tested with an unfamiliar partner showed less rearing (permuted *t* test, *t* = 2.3638, df = 34, *P* = 0.0069) than animals tested with a familiar partner, and rats tested with a partner of a different strain presented less prosocial behavior than rats tested with a familiar (Fig. 4C-4, permuted *t* test, *t* = 3.3195, df = 34, *P* = 0.0029) or an unfamiliar partner of the same strain (permuted *t* test, *t* = 2.5997, df = 22, *P* = 0.0087). For other statistical comparisons, see Table S2. Further, we recorded less 50-kHz USV in rats tested with unfamiliar partner than in rats tested with familiar partner (Fig. 4C-5, permuted *t* test, *t* = 2.4341, df = 28, *P* = 0.0129). Together, these results show that the most effective buffers of fear during fear extinction are either familiar partners subjected to extensive fear extinction or naive animals. In our further research, we used the familiar fear-extinguished partners as they had a similar history of exposure to procedures used in the experiments as the tested animals.

### Extinction with a partner strongly inhibits fear responses, but it is insufficient to improve fear extinction memory

To test the persistence of fear extinction memory following accompanied extinction, we subjected rats to an extinction session either with a partner or alone (Extinction Test 1, Fig. 5A). We then tested their freezing response during 2 subsequent solitary extinction sessions (Extinction Tests 2, 3, Fig. 5A). The level of freezing at the end of the fear conditioning session was similar in both groups (Fig. 5B, inserted graph, the freezing averaged across the last 2 CS: Mann-Whitney test, *U* = 46.5, *P* = 0.2407). The freezing level during the extinction session diminished in rats tested together (Fig. 5A, the freezing averaged across the last 2 CS compared with averaged freezing to the CS during extinction test 1: paired *t* test, *t* = 5.888, df = 11, *P* = 0.0001), but not in rats tested alone (Wilcoxon matched-pairs signed rank test, *W* = 20, *P* = 0.4131).

Next, we compared averaged freezing to the CS, freezing to the subsequent CS, other behavioral measures, and USVs between rats tested with a partner and rats tested alone during all 3 extinction tests and then proceeded with planned comparisons between the groups. There were differences in averaged freezing to the CS (Fig. 5B, permuted ANOVA, *F*(5, 56) = 18.7961, *P* < 0.0001) and in freezing in response to the subsequent CS [Fig. 5C, mixed ANOVA, group: *F*(5, 930) = 19.8905, *P* < 0.0001, time: *F*(15, 930) = 18.7450, *P* < 0.0001, group x time: *F*(15, 930) = 2.5822, *P* < 0.0001]. Further, the analysis showed differences in quiescence [permuted ANOVA, *F*(5, 56) = 32.3331, *P* < 0.0001], mesh sniffing [permuted ANOVA, *F*(5, 56) = 11.1540, *P* < 0.0001], prosocial [permuted ANOVA, *F*(5, 56) = 17.2359, *P* < 0.0001], rearing [permuted ANOVA, F(5, 56) = 4.1215, *P* = 0.0021], number of 22-kHz USVs [permuted ANOVA, F(5, 53) = 14.7478, *P* < 0.0001], and number of 55-kHz USVs [permuted ANOVA, *F*(5, 55) = 14.5672, *P* < 0.0001], but not in cage exploration [permuted ANOVA, *F*(5, 56) = 1.6724, *P* = 0.1594].

Then, we proceeded with planned comparisons between groups. As before, the rats tested with a partner showed potent inhibition of fear [Fig. 5B, extinction test 1, average freezing level to the CS, permuted *t* test, *t* = 7.7490, df = 19, *P* < 0.0001; Fig. 5C, extinction test 1, freezing in response to the subsequent CS, mixed ANOVA, group: *F*(1, 315) = 72.9083, *P* < 0.0001, time: *F*(15, 315) = 12.9554, *P* < 0.0001, group x time: *F*(15, 315) = 3.6804, *P* < 0.0001]. Subjects tested with partners performed more mesh sniffing (Fig. 5D, extinction test 1, permuted *t* test, *t* = 4.9285, df = 19. *P* < 0.0001), and more prosocial behavior (Fig. 5D, extinction test 1, permuted *t* test, *t* = 4.1141, df = 19, *P* < 0.0001) in comparison to the animals tested alone that spend much more time motionless (Fig. 5D, extinction test 1, quiescence: permuted *t* test, *t* = 8.9653, df = 19, *P* < 0.0001). The rats also produced much less 22-kHz USVs (Fig. 5E, permuted *t* test, *t* = 6.3211, df = 21, *P* < 0.0001) and more 50-kHz USVs (Fig. 5E, permuted *t* test, *t* = 4.2110, df = 21, *P* < 0.0001) when tested with a partner than the animals subjected to solitary extinction. However, when tested alone the next day (extinction test 2), the animals showed similar freezing levels as rats subjected to non-accompanied fear extinction [Fig. 5B, average freezing level to the CS, permuted *t* test, *t* = 7.7490, df = 19, not significant after FDR correction; Fig. 5C, freezing in response to the subsequent CS, mixed ANOVA, group: *F*(1, 315) = 0.0190, not significant after FDR correction, time: *F*(15, 315) = 10.5621, *P* < 0.0001, group x time: *F*(15, 315) = 2.5040, *P* = 0.0017]. Even if the level of freezing of rats subjected to accompanied fear extinction was slightly lower than in rats subjected to non-accompanied fear extinction at the beginning of the extinction session, it was significantly higher than the day before when tested with a partner [Fig. 5B, extinction test 1, 2, average freezing level to the CS, permuted *t* test, *t* = 4.0114, df = 20, *P* = 0.0011; Fig. 5C, extinction tests 1, 2, freezing in response to the subsequent CS, mixed ANOVA, group: *F*(1, 330) = 13.9625, *P* = 0.0010, time: *F*(15, 330) = 7.4746, *P* < 0.0001, group x time: *F*(15, 330) = 4.0843, *P* < 0.0001]. Similarly, the groups did not differ in the level of mesh sniffing or quiescence, in extinction tests 2 (Fig. 5D, extinction test 2, mesh sniffing: permuted *t* test, *t* = 0.6441, df = 19, not significant after FDR correction; quiescence: permuted *t* test, *t* = 0.6087, df = 19, not significant after FDR correction). The number of 20-kHz and 50-kHz USVs produced also did not differ between the groups (Fig. 5D, extinction test 2, number of 22-kHz USVs, permuted *t* test, *t* = 1.5796, df = 17, not significant after FDR correction; number of 55-kHz USVs, permuted *t* test, *t* = 0.7946, df = 17, not significant after FDR correction). Number of 22-kHz USVs was significantly higher (Fig. 5E, extinction tests 1 and 2, permuted *t* test, *t* = 4.0723, df = 19, *P* = 0.0011), while number of 50-kHz USVs was significantly lower (Fig. 5E, extinction tests 1 and 2, permuted *t* test, *t* = 3.7127, df = 19, *P* < 0.0001) than during the accompanied extinction session. On the following day of testing (extinction test 3), we also did not observe any differences in the level of freezing [Fig. 5B, average freezing level to the CS, permuted *t* test, *t* = 0.0106, df = 18, not significant after FDR correction; Fig. 5C, freezing in response to the subsequent CS, mixed ANOVA, group: *F*(1, 300) = 0.2865, not significant after FDR correction, time: *F*(15, 300) = 3.5108, *P* < 0.0001, group x time: *F*(15, 300) = 1.1338, not significant after FDR correction], and also in mesh sniffing and quiescence (Fig. 5D, extinction test 3, mesh sniffing: permuted *t* test, *t* = 2.3243, df = 0.0320, not significant after FDR correction; quiescence: permuted *t* test, *t* = 0.2766, df = 18, not significant after FDR correction). The number of both, 22-kHz and 55-kHz USVs also did not differ between the groups (Fig. 4E, extinction test 3, number of 22-kHz USVs, permuted *t* test, *t* = 1.8201, df = 15, not significant after FDR correction; number of 50-kHz USVs, permuted *t* test, *t* = 1.0570, df = 15, not significant after FDR correction). For the comparisons between extinction tests 1, 2, and 3, please see Table S2. It is worth noting that the visual inspection of the video recordings revealed that at the end of extinction tests 2 and 3 the rats presented a relaxed posture, which was classified as freezing by the automated analysis. As such it appears in the graphs as increased freezing. In sum, when rats were tested alone following accompanied extinction, they displayed significantly higher freezing levels to CS than when tested with a partner.

### Projections from the anterior cingulate cortex to the central amygdala (ACC–CeA) are involved in social inhibition of fear during extinction

**Fig. 6:**
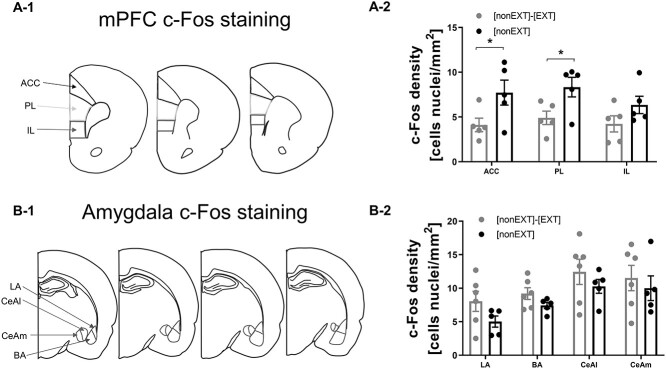
Social buffering decreases c-Fos expression in the ACC and PL. (A-1) Scheme of coronal sections used for measuring c-Fos activation in the prefrontal cortex. (A-2) Fear extinction in pairs ([nonEXT]-[EXT], *n* = 5) resulted in decreased activation of the ACC and the PL compared to animals subjected to unaccompanied fear extinction ([nonEXT], *n* = 5), but there were no differences in activation of the IL. (B-1) Scheme of coronal sections used for measuring c-Fos activation in the amygadala. (B-2) The accompanied ([nonEXT]-[EXT], *n* = 5) extinction did not change the neuronal activation pattern of the lateral (LA) and basal (BA) nucleus of amygdala and lateral (CeAl) and medial (CeAm) division of the central nucleus amygdala in comparison with unaccompanied fear extinction ([nonEXT], *n* = 5). The graphs show means ± SEM, ^*^P < 0.05.

To compare activation of the brain during the accompanied and unaccompanied fear extinction test, we mapped c-Fos expression in brain slices of subject rats collected 90 min after exposure to the first CS of extinction test 1. We focused on the prefrontal cortex (Fig. 6A-1), the structure involved in fear extinction. We found decreased activation of the ACC, specifically A24 (van Heukelum et al. 2020) and PL in rats tested with a partner compared to the animals tested alone [Fig. 6A-2, 2-way ANOVA, *F*(1,24) = 13.92, *P* = 0.001, FDR test, ACC: *q* = 0.0354, PL: *q* = 0.0354]. In contrast, there was no difference in the level of activation of the infralimbic cortex (IL, FDR test, *q* = 0.1474). In the amygdala, we observed slightly higher level of c-Fos expression in rats tested with a partner [Fig. 6B-1, B-2, 2-way ANOVA, *F*(1, 36) = 4.281, *P* = 0.0458], but the FDR test did not reveal significant changes in any of the tested amygdalar nuclei (the lateral, LA: *q* = 0.4634, basal, BA, *q* = 0.4634, central medial, CeM, *q* = 0.4634, and central lateral, CeAl, *q* = 0.4634) compared to the animals tested alone.

**Fig. 7:**
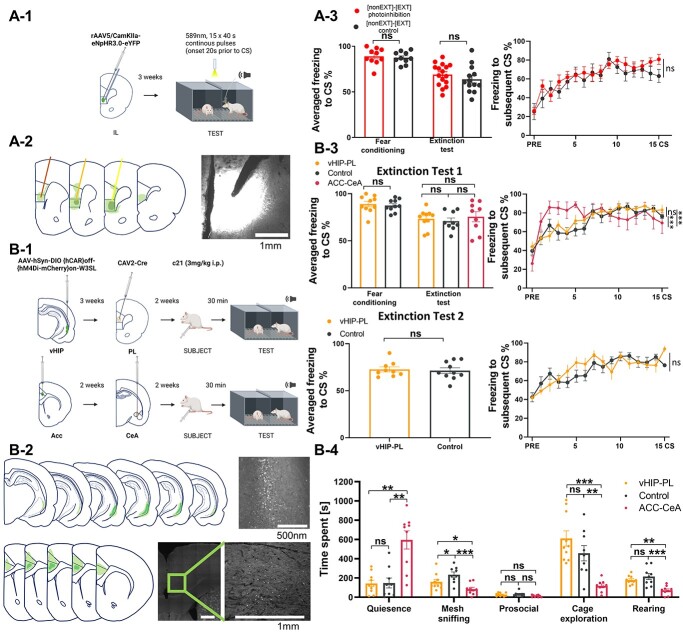
Modulation of social buffering by projections from the anterior cingulate cortex to the central amygdala (ACC–CeA). (A1) Scheme of optoinhibition of the IL of the subject rat during extinction test with partner. (A2) Localization of viral expression and optic fiber placement in the IL: schematic mark-up and an example image. (A3) Photoinhibition ([nonEXT]-[EXT]photoinhibition, *n* = 16) of the IL during accompanied extinction did not change the freezing level in comparison with control animals ([nonEXT]-[EXT]control, *n* = 13). (B1) Scheme of chemoinhibition of vHIP–PL pathway (upper panel) and ACC–CeA pathway (lower panel). (B2) Localization of viral expressions in the vHip (upper panel) and in the ACC (lower panel) with example images. (B3) Extinction test 1. Inhibition of the ACC–CeA (ACC–CeA, red, *n* = 9) but not vHIP (vHIP–PL, yellow, *n* = 10) reduced social buffering in comparison with control (control, black, *n* = 9). Left graph represents averaged freezing level during 2 last CS of fear conditioning (for ACC–CeA data had been lost due to technical problems with recording) and averaged freezing to the CS during extinction test of each rat; right graph represents freezing levels to the subsequent CS during extinction test. Extinction test 2. Chemoinhibition (vHIP–PL, yellow, *n* = 10) of the vHIP–PL pathway did not change freezing during accompanied extinction in comparison with control (control, black, *n* = 10). (B-4) Graphs show the duration of different behaviors throughout the first accompanied extinction session (extinction test 1). Chemoinhibition of the ACC–CeA pathway (ACC–CeA, red, *n* = 9) increased the quiescence and reduced mesh sniffing and cage exploration in comparison with control (control, black, *n* = 9). Inhibition of the vHIP–PL pathway (vHIP–PL, yellow, *n* = 10) decreased mesh sniffing. The graphs show means ± SEM, ^*^P < 0.05, ^*^^*^P < 0.01, ^*^^*^^*^P < 0.001.

Next, we tested the function of the IL in accompanied fear extinction using loss-of-function approach (Fig. 7). As the IL has been implicated before in fear extinction (Quirk et al. 2000; Do-Monte et al. 2015; Brill-Maoz and Maroun 2016) and social buffering (Brill-Maoz and Maroun 2016), we tested the effects of the IL optogenetic inhibition (Fig. 7A-1, A-2) on the accompanied and unaccompanied fear extinction (Do-Monte et al. 2015). Both control and optoinhibited groups had the same level of freezing at the end of the fear conditioning session (Fig. 7A-3 left panel, the freezing averaged across the last 2 CS, Mann-Whitney test, *U* = 91, *P* = 0.5804). We did not observe any effects of the IL inhibition on the level of freezing during the accompanied fear extinction (Fig. 7A-3, left panel: average freezing level to the CS, *t* test, *t* = 1.385, df = 32, *P* = 0.1757; right panel: freezing in response to the subsequent CS, 2-way ANOVA, group: *F*(1, 27) = 0.8330, *P* = 0.3695; group x CS: *F*(15, 405) = 0.6818, *P* = 0.8030). Thus, we focused on 2 pathways in the following steps, the vHIP–PL and ACC–CeA, which our previous studies implicated in fear extinction ([Bibr ref47][Bibr ref47]) and social interaction (Andraka et al. 2021), respectively.

We observed before that the vHIP–PL projection suppresses fear response after prolonged fear extinction, gradually changing their role as fear extinction training progresses, initially promoting and then attenuating fear response (Szadzinska et al. 2021). In contrast, the vHIP–BL projection is activated when the freezing level is high (Sotres-Bayon et al. 2012; Szadzinska et al. 2021). Functional projections tracing showed that activated cells in the PL of rats subjected to fear extinction in pairs receive more prominent input from the vHIP (Fig. S1) than from the BL. Thus, we hypothesized that the vHIP–PL pathway could also be involved in accompanied fear extinction.

To check the role of the vHIP–BL projection in the social buffering effect, we inhibited this pathway during accompanied extinction. We tested inhibition of the vHIP–PL pathway twice, during 2 subsequent accompanied fear extinction sessions, as we expected gradual changes during the training (Szadzinska et al. 2021). We used the chemogenetic construct that allows for efficient inhibition of the projection (Li et al. 2018). The control rats expressed the same construct, but we injected them with saline instead of injecting the C21 compound, which activates the construct.

We injected half of the animals with the C21 compound before the first session and saline before the second session, the other half with saline before the first session, and the C21 compound before the second session (Fig. 7B-1). Both groups, with vHIP–PL pathway inhibited and control group, had the same level of freezing at the end of the fear conditioning session (Fig. 7B-3, left panel, the freezing averaged across the last 2 CS, *t* test, *t* = 0.4256, df = 18, *P* = 0.6755). Then, we compared behavioral measures of the groups from the chemogenetic experiments, during extinction tests 1 and 2 (Fig. 7B-3). Analysis of the average freezing levels to the CS revealed no difference between the groups [permuted ANOVA, *F*(4, 37) = 0.5150, *P* = 0.72108]. However, there was a difference in freezing in response to the subsequent CS [mixed ANOVA revealed no effect of group: *F*(4, 630) = 0.2589, not significant after FDR correction, but strong effect of time: *F*(15, 630) = 25.7847, *P* < 0.0001 and interaction between those 2 factors: (*F*60, 630) = 2.0546, *P* < 0.0001]. Analysis of other behavioral measures showed a significant differences in quiescence: [permuted ANOVA, *F*(2, 22) = 13.3532, *P* = 0.0004], mesh sniffing [permuted ANOVA, *F*(2, 22) = 10.9816, *P* = 0.0007], prosocial [permuted ANOVA, *F*(2, 22) = 3.5890, *P* = 0.0321], cage exploration [permuted ANOVA, *F*(2, 22) = 11.3547, *P* = 0.0005], and rearing [permuted ANOVA, *F*(2, 22) = 12.1467, *P* = 0.0002]. We then proceeded with planned comparisons between the groups.

We observed no difference in the freezing level between the group with inhibited vHIP–PL pathway and control group during the first extinction session [Fig. 7B-3, Extinction Test 1, left panel, average freezing level to the CS, permuted *t* test, *t* = 0.7068, df = 15, not significant after FDR correction; right panel, freezing in response to the subsequent CS, mixed ANOVA, group: *F*(1, 255) = 0.2719, not significant after FDR correction, time: *F*(15, 255) = 25.7848, *P* < 0.0001, group x time: *F*(15, 255) = 0.6956, not significant after FDR correction]. We also found no difference in the freezing level during the second session [Fig. 7B-3, extinction test 2, left panel, average freezing level to the CS, permuted *t* test, *t* = 0.2026, df = 15, not significant after FDR correction; right panel, freezing in response to the subsequent CS, mixed ANOVA, group: *F*(1, 255) = 0.1077, not significant after FDR correction; time: *F*(15, 255) = 12.5154, *P* < 0.0001; time x group: *F*(15, 255) = 1.8301, not significant after FDR correction] of accompanied extinction between the vHIP–PL and control groups. We only observed the decreased mesh sniffing behavior after inhibition of the vHIP–PL pathway during the first testing session (Fig. 7B-4, permuted *t* test, *t* = 2.3358, df = 15, *P* = 0.0299). Together, the results suggest that there is no effect of the vHIP–PL pathway on social buffering during the first 2 sessions of fear extinction.

As manipulating with activity of the structures or pathways implicated earlier in solitary fear extinction showed no significant effects on social buffering, next we focused on another pathway. Our current data suggested that fear inhibition during accompanied fear extinction strongly depends on social factors. The ACC (Han et al. 2019), and the ACC–CeA pathway (Andraka et al. 2021) have been previously implicated in social interaction with a fearful partner. Thus, we inhibited the ACC–CeA projection during accompanied extinction. We observed a significantly reduced social buffering effect. Freezing response to CS increased after the first CS, and the high freezing level remained elevated throughout the testing session. It was not visible in the averaged freezing to the CS (Fig. 7B-3, left panel, permuted *t* test, *t* = 0.4174, df = 15, not significant after FDR correction), but in freezing in response to the CS [Fig. 7B-3, right panel, mixed ANOVA revealed no significant effect of group: *F*(1, 240) = 0.5557, not significant after FDR correction, but highly significant effects of time: *F*(15, 240) = 8.4108, *P* < 0.0001, and interaction between group and time: *F*(15, 240) = 3.1105, *P* = 0.0001]. The effect of inhibition of the ACC–CeA pathway on freezing was also different from blocking the vHIP–PL pathway [Fig. 7B-3, left panel, averaged freezing to CS, permuted *t* test, *t* = 0.8248, df = 14, not significant after FDR correction; freezing in response to the CS, Fig. 7B-3, right panel, mixed ANOVA, group: *F*(1, 255) = 0.1406, not significant after FDR correction; time: *F*(5, 255) = 8.6901, *P* < 0.0001; group x time: *F*(1, 255) = 3.4155, *P* < 0.0001]. A more detailed analysis of rats’ behavior during the whole extinction test session revealed that the rats with blocked ACC–CeA pathway significantly decreased mesh sniffing, cage exploration and rearing, in comparison to the control group (Fig. 7B-4, mesh sniffing, permuted *t* test, *t* = 5.2064, df = 14, *P* < 0.0001, cage exploration, permuted *t* test, *t* = 3.8101, df = 14, *P* = 0.0089, rearing, permuted *t* test, *t* = 4.2019, df = 14, *P* < 0.0001), as well as in comparison to the vHIP–PL group (Fig. 7B-4, mesh sniffing, permuted *t* test, *t* = 2.2852, df = 15, *P* = 0.0321, cage exploration, permuted *t* test, *t* = 5.0920, df = 15, *P* < 0.0001, rearing, permuted *t* test, *t* = 4.7463, df = 15, *P* = 0.0007). The rats from the ACC–CeA group also increased time spent immobile in comparison to the control group (Fig. 7B-4, quiescence: permuted *t* test, *t* = 3.8090, df = 14, *P* = 0.0089) and to the vHIP–PL group (Fig. 7B-4, quiescence: permuted *t* test, *t* = 4.1037, df = 15, *P* = 0.0029). For other statistical comparisons, see Table S2.

To check the impact of chemoinhibition on rats’ locomotor activity and anxiety, we performed the open field test on the subset of animals. The locomotor activity was not changed by the ACC–CeA inhibition (ctrl: 11,961 ± 7760; C21: 22,119 ± 11,181, Welch’s *t* test, *t* = 1.669, df = 7.128, *P* = 0.1383). However, the number of entries to the center of the open field was different between the groups (ctrl: 3.4 ± 2.702; C21: 7.8 ± 3.114, unpaired *t* test, *t* = 2.386, df = 8, *P* = 0.0441). The animals with inhibited the ACC–CeA pathway performed more entries to the center. However, the average time of each entry in this group was shorter (ctrl: 8.482 ± 4.258; C21: 2.846 ± 1.36, Welch’s *t* test, *t* = 2.819, df = 4.807, *P* = 0.0388), and average velocity upon entry was higher (ctrl: 2.361 ± 0.2756; C21: 3.604 ± 0.6871, Mann-Whitney test, *U* = 1, *P* = 0.0159) than in the control group. The results suggest the change in exploration pattern in the rats with blocked ACC–CeA pathway, but do not show inhibition of exploratory activity.

## Discussion

Here we show that the presence of a partner rat during fear extinction decreases fear responses. However, although social inhibition of fear is very robust, the effect is transient and disappears when rats are tested individually on the next day. We found that social inhibition of fear is more potent in familiar and same strain animals. It also depends on the emotional status of the partner. The fear-conditioned partners were less effective at decreasing the subjects’ fear than those subjected earlier to fear extinction. At the neuronal level, we observed decreased activation of the PL and ACC cortices but not the IL in animals subjected to fear extinction with a companion as compared with animals tested alone. In line with the mapping results, photoinhibition of the IL left social inhibition of fear intact. Similarly, inhibition of the vHIP–PL pathway, which suppresses fear response after prolonged solitary extinction training (Szadzinska et al. 2021), did not diminish social buffering of fear. We then tested the ACC–central amygdala (ACC–CeA) pathway, as this cortical input to the amygdala is involved in socially mediated information transfer (Allsop et al. 2018; Andraka et al. 2021). The inhibition of the ACC–CeA significantly decreased social contact and blocked social buffering of fear during extinction.

Previous studies showed that the presence of a conspecific reduces fear and attenuates activation of several brain structures involved in fear response, including the prefrontal cortex and amygdala (Davitz and Mason 1955; Kiyokawa et al. 2007; Fuzzo et al. 2015). However, we know little about social inhibition of fear during extinction and the neuronal circuits involved. Earlier studies showed that the presence of a familiar conspecific during fear extinction inhibits freezing response (Brill-Maoz and Maroun 2016; Mikami et al. 2016; Ferreira et al. 2019), and implicated the prefrontal cortex in this inhibition (Brill-Maoz and Maroun 2016; Penha Farias et al. 2019). The inhibition of freezing by partner’s presence was interpreted as facilitation of extinction. However, freezing suppression may result from social interaction or a change of the experimental context. We aimed at clarifying this issue by testing the persistence of social buffering effects and the effect of context change.

We found that though social inhibition of fear is very robust, it is also transient. The previously published results on the long-term effects of accompanied fear extinction are not consistent. Mikami et al. (2016, 2020) have reported decreased fear response on the day following the accompanied extinction session. The discrepancy between their results and ours may stem from diverse severity of the aversive stimulation. They used weaker foot shocks, which resulted with lower levels of conditioned fear responses than what we observed in our study. We aimed at modeling trauma and used a more robust protocol, resulting with strong fear responses. In the study by Brill-Maoz and Maroun (2016), the reinstatement test showed that a companion’s presence during extinction (which effectively inhibited freezing), did not prevent reinstatement of fear when they tested the rats separately. Similarly, in the study by Penha Farias et al. (2019), extinction memory was not affected by the presence of a familiar non-fearful conspecific during extinction training. Our results show that one session of accompanied extinction has no effect on retrieval of fear memory on the next day. Although inhibition of fear when another rat is present is clear, it does not significantly affect the fear memory tested individually on the following day.

Since extinction is strongly context-dependent (Bouton et al. 2006), fear inhibition could occur if the rats perceived the “pair context” as dissimilar to the conditioning context. To test this hypothesis, we conditioned rats together and then tested them in the same social context, i.e. with the partner with whom they were conditioned. We did observe a decrease in the social buffering effect, but the level of freezing was still much lower than in rats tested separately. Thus, we showed that the context change is not sufficient to explain the social buffering effect we observed. In line, our results also indicate that social buffering depends on social factors, familiarity, and emotional state of a partner rat. We show that, during fear extinction test, partner rats not subjected to fear extinction do not inhibit fear of the subjects as well as partners subjected to fear extinction or naïve ones. Moreover, subjects appear to interact more with the fear extinguished partner than with the naïve partner. This might be due to a different emotional status of the naïve, previously not conditioned partner (Scheggia et al. 2020). Our data also show that familiar and same strain partners are more effective at inhibiting fear than non-familiar or different-strain animals. These results are consistent with previous reports on the effect of a partner’s emotional state (Kiyokawa et al. 2004), and familiarity (Kiyokawa et al. 2009) during pair exposure to fear-inducing stimuli. One of the postulated mechanisms of social reduction of fear is distracting attention from the threatening environment (Epley 1974). As novel physical or social object attracts more attention than familiar ones, if the social buffering effects we observed were related to a distraction of attention, we could expect more potent effects in rats tested with unfamiliar partners. In contrast, we observed that rats’ familiarity and physical similarity positively modulate social buffering during fear extinction. Thus, it seems more plausible that it is a socially specific effect rather than just a distraction of attention.

One of the factors we did not investigate is the sex of the animals. There is one report about social buffering of conditioned fear response in female rats (Ishii et al. 2016) showing that estrus cycle affected neither intensity of fear response nor response to conspecific presence. Nevertheless, this is a factor which requires further studies, as sex differences in social behaviors of rodents are commonly observed (Stack et al. 2010), and anxiety (Olff 2017) disorders have higher prevalence in females (McLean et al. 2011).

To test whether social inhibition of fear shares neuronal mechanisms with individual fear extinction, we measured activation of fear regulating neuronal circuits. The infralimbic part of the medial prefrontal cortex (IL) orchestrates Pavlovian extinction (Morgan et al. 1993; Quirk et al. 2000; Do-Monte et al. 2015), while the PL, vHIP, and BL have been strongly implicated in extinction learning (Maren 2011). A recent report showed that social inhibition of fear relies on the ventromedial prefrontal cortex (Penha Farias et al. 2019). Thus, we focused on different parts of the prefrontal cortex and their inputs from the vHIP and BL. Our data show that social inhibition of fear only partially shares the neuronal circuits of individual fear extinction. The IL inhibition did not affect social buffering. This is in contrast to the results of Brill-Maoz and Maroun (2016) who showed that infusion of oxytocin antagonist to the IL disrupts social buffering during fear extinction. The disparity may stem from differences in the behavioral procedure or from the fact that we inhibited the neurons within the IL non-specifically, whereas in the study of Brill-Maoz et al. oxytocin receptors were selectively blocked.

Similarly, inhibition of the vHIP–PL pathway did not increase freezing suggesting that this pathway is not involved in social inhibition of fear. Our previous study implicated the vHIP–PL pathway in individual fear extinction. We showed that the vHIP–PL pathway gradually changes its role as fear extinction training progresses, initially promoting and then attenuating the fear response (Szadzinska et al. 2021). In line, here, we observed activation of the vHIP–PL pathway when a partner’s presence lowered the level of fear. However, chemogenetic inhibition of this pathway did not affect a CS-specific increase of freezing response, i.e. it did not diminish the social buffering effect. The result shows that, at least during the first 2 sessions of accompanied extinction, the pathway is not causally involved in social buffering effect.

As the behavioral results suggested that fear inhibition depends strongly on the social component, we focused on the pathway implicated in social behavior. Notably, the ACC and its projection to the amygdala process socially derived threat information rather than mediate conditioned fear response (Jeon et al. 2010; Allsop et al. 2018). Similarly, our earlier results implicated the ACC–CeA pathway in socially transferred fear (Andraka et al. 2021). We observed almost complete blocking of the social buffering by inhibition of the ACC–CeA pathway. The freezing was increased after the first CS and stayed very high throughout the whole extinction session. The ACC–CeA pathway blocking also diminished social contacts with a partner, which increased in all instances of adequate social buffering in this study. Notably, the basal level of freezing (before the first CS) was not changed, which shows that the ACC–CeA pathway modulates responses to fear-evoking stimuli rather than affects the level of contextual fear.

The transient effect of social inhibition of fear echoes the clinical pitfalls of using safety signals, i.e. predictors of the absence of the US such as the presence of another person, therapists, medications, food, or drink (Craske et al. 2008). The safety signals alleviate fear response in the short term but do not affect the fear-evoking properties of the CS subsequently tested without the safety signal. The transient effects of safety signals have been explained by their association with the absence of an expected aversive outcome; forming such an association with safety signals rather than with the CS does not improve extinction. The safety signals have also been suggested to promote avoidance of fear processing, which interferes with the development of new, non-threat associations necessary for successful fear extinction (Craske et al. 2008). Our data do not support this notion; although the presence of another individual did not improve fear extinction, it also did not impede it.

In sum, our data show that social buffering of fear is transient and only partially shares neuronal circuits with individual fear extinction. Instead, the involvement of ACC–amygdala projection, implicated earlier in modulation of social behavior, makes it a good candidate for potential therapeutic interventions. Whether local manipulation of circuit activity with transcranial magnetic stimulation or transcranial direct current stimulation could aid exposure based therapies and prolong the effect of social buffering in humans remains to be determined.

Abbreviations
ACC: anterior cingulate cortex
BA: basal nucleus of amygdala
BL: abasolateral nucleus of the amygdala
CeAm: medial part of central nucleus of the amygdala
CeAl: lateral part of central nucleus of the amygdala
CS: conditioned stimulus
FDR: false discovery rate
IL: infralimbic cortex
LA: lateral nucleus of amygdala
PL: prelimbic cortex
USV: ultrasonic vocalizations
US: unconditioned stimulus
vHIP: ventral hippocampus

## Supplementary Material

Figure_S1_bhac395

Table_S1_bhac395

Table_S2_bhac395

suplementary_materials_EK_bhac395
