# Supplementary figures and images for: Social buffering diminishes fear response but does not equal improved fear extinction
Source: Cereb Cortex. 2022 Oct 11;33(8):5007–24. doi: 10.1093/cercor/bhac395 (PMC10110450; doi:10.1093/cercor/bhac395)

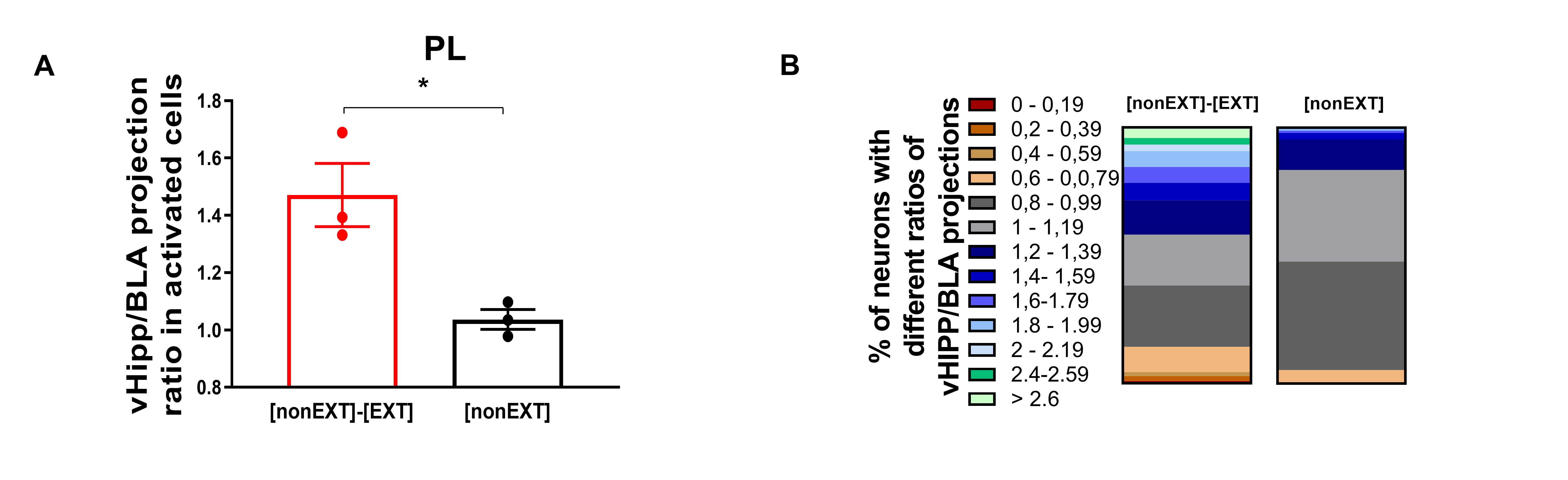

Supplement: Figure_S1_bhac395 [file figure_s1_bhac395.jpeg]
