# Supplementary material for: Social buffering diminishes fear response but does not equal improved fear extinction
Source: Cereb Cortex. 2022 Oct 11;33(8):5007–24. doi: 10.1093/cercor/bhac395 (PMC10110450; doi:10.1093/cercor/bhac395)
Supplement: Table_S1_bhac395 [file table_s1_bhac395.docx]

**Table 1**. Description of the experimental groups.

| Group name | Subject (S) /Partner (P) | Day 1 | Day 2-4 | Day 5 | strain/  familiarity | partition | Figure |
| --- | --- | --- | --- | --- | --- | --- | --- |
| [nonEXT]- [EXT] | S - [nonEXT] | fear conditioning alone | cage exposure alone | fear extinction with a partner | Wistar/ familiar | wire mesh | 1 |
|  | P - [EXT] | fear conditioning alone | fear extinction alone | fear extinction with a partner | Wistar/ familiar | wire mesh | 1 |
| [nonEXT] | S - [nonEXT] | fear conditioning alone | cage exposure alone | fear extinction alone | Wistar | wire mesh | 1 |
| [nonEXT]-[ EXT] - conditioned together | S - [nonEXT]- conditioned together | fear conditioning together | cage exposure alone | fear extinction with a partner | Wistar/ familiar | wire mesh | 2A |
|  | P - [EXT] - conditioned together | fear conditioning together | fear extinction alone | fear extinction with a partner | Wistar/ familiar | wire mesh | 2A |
| [nonEXT]-[anesthetized rat] | S - [nonEXT] | fear conditioning alone | cage exposure alone | fear extinction with a partner | Wistar/ familiar | wire mesh | 2B |
|  | P -[anesthetized rat] | fear conditioning alone | cage exposure alone | anesthetized | Wistar/ familiar | wire mesh | 2B |
| [nonEXT]- [EXT]-no visual cues | S - [nonEXT] | fear conditioning alone | cage exposure alone | fear extinction with a partner | Wistar/ familiar | non-transparent | 2C |
|  | P - [EXT]-no visual cues | fear conditioning alone | fear extinction alone | fear extinction with a partner | Wistar/ familiar | non-transparent | 2C |
| [nonEXT]- [nonEXT] | S - [nonEXT] | fear conditioning alone | cage exposure alone | fear extinction with a partner | Wistar/ familiar | wire mesh | 4A |
|  | P - [nonEXT] | fear conditioning alone | cage exposure alone | fear extinction with a partner | Wistar/ familiar | wire mesh | 4A |
| [nonEXT]-[naive partner] | S - [nonEXT] | fear conditioning alone | cage exposure alone | fear extinction with a partner | Wistar/ familiar | wire mesh | 4B |
|  | P - [naive partner] | cage exposure alone | cage exposure alone | cage exposure with a partner | Wistar/ familiar | wire mesh | 4B |
| [nonEXT]-[unfamiliar rat] | S - [nonEXT] | fear conditioning alone | cage exposure alone | fear extinction with a partner | Wistar/ unfamiliar | wire mesh | 4C |
|  | P - [unfamiliar rat] | cage exposure alone | cage exposure alone | cage exposure with a partner | Wistar/ unfamiliar | wire mesh | 4C |
| [nonEXT]-[other strain rat] | S - [nonEXT] | fear conditioning alone | cage exposure alone | fear extinction with a partner | Wistar/ unfamiliar | wire mesh | 4C |
|  | P - [other strain rat] | cage exposure alone | cage exposure alone | cage exposure with a partner | Long-Evans/ unfamiliar | wire mesh | 4C |
