# Supplementary material for: Social buffering diminishes fear response but does not equal improved fear extinction
Source: Cereb Cortex. 2022 Oct 11;33(8):5007–24. doi: 10.1093/cercor/bhac395 (PMC10110450; doi:10.1093/cercor/bhac395)
Supplement: Table_S2_bhac395 [file table_s2_bhac395.docx]

**Table 2**. Statistics.

| **Figure** | **Variable** | **Groups** | **n** | **Test** | **Test statistics** | **Significance** |
| --- | --- | --- | --- | --- | --- | --- |
| **Figure 1** | | | | | | |
| 1B-3 | cage exploration | [nonEXT]-[EXT] vs [nonEXT] | 24 vs 23 | permuted t test | t=1.3713, df=44 | not significant after FDR correction |
| **Figure 2** | | | | | | |
| 2A-4 | quiescence | [nonEXT]-[EXT]-conditioned together vs [nonEXT]-[ EXT] | 12 vs 24 | permuted t test | t=1.2072, df=34 | not significant after FDR correction |
| 2A-4 | mesh sniffing | [nonEXT]-[EXT]-conditioned together vs [nonEXT]-[ EXT] | 12 vs 24 | permuted t test | t=0.583, df=34 | not significant after FDR correction |
| 2A-4 | cage exploration | [nonEXT]-[EXT]-conditioned together vs [nonEXT]-[ EXT] | 12 vs 24 | permuted t test | t=2.0077, df=34 | not significant after FDR correction |
| 2A-4 | rearing | [nonEXT]-[EXT]-conditioned together vs [nonEXT]-[ EXT] | 12 vs 24 | permuted t test | t=1.3583, df=34 | not significant after FDR correction |
| 2B-4 | mesh sniffing | [nonEXT]-[anaesthetized rat] vs [nonEXT]-[EXT] | 6 vs 24 | permuted t test | t=1.0323, df=28 | not significant after FDR correction |
| 2B-4 | cage exploration | [nonEXT]-[anaesthetized rat] vs [nonEXT]-[EXT] | 6 vs 24 | permuted t test | t=3.3186, df=28 | 0.0051 |
| 2B-4 | rearing | [nonEXT]-[anaesthetized rat] vs [nonEXT]-[EXT] | 6 vs 24 | permuted t test | t=1.8388, df=28 | not significant after FDR correction |
| 2C-4 | quiescence | [nonEXT]-[EXT]-visual cues vs [nonEXT]-[EXT] | 11 vs 24 | permuted t test | t=0.8121, df=33 | not significant after FDR correction |
| 2C-4 | mesh sniffing | [nonEXT]-[EXT]-visual cues vs [nonEXT]-[EXT] | 11 vs 24 | permuted t test | t=1.9167, df=33 | not significant after FDR correction |
| 2C-4 | prosocial behavior | [nonEXT]-[EXT]-visual cues vs [nonEXT]-[EXT] | 11 vs 24 | permuted t test | t=0.7981, df=33 | not significant after FDR correction |
| 2C-4 | cage exploration | [nonEXT]-[EXT]-visual cues vs [nonEXT]-[EXT] | 11 vs 24 | permuted t test | t=2.9148, df=33 | p=0.0031 |
| 2C-4 | rearing | [nonEXT]-[EXT]-visual cues vs [nonEXT]-[EXT] | 11 vs 24 | permuted t test | t=1.1410, df=33 | not significant after FDR correction |
| **Figure 4** | | | | | | |
| 4A-4 | quiescence | [nonEXT]-[nonEXT] vs [nonEXT]-[EXT] | 24 vs 24 | permuted t test | t=4957, df=46 | not significant after FDR correction |
| 4A-4 | mesh sniffing | [nonEXT]-[nonEXT] vs [nonEXT]-[EXT] | 24 vs 24 | permuted t test | t=1.7721, df=46 | not significant after FDR correction |
| 4A-4 | prosocial behavior | [nonEXT]-[nonEXT] vs [nonEXT]-[EXT] | 24 vs 24 | permuted t test | t=0.2976, df=46 | not significant after FDR correction |
| 4A-4 | cage exploration | [nonEXT]-[nonEXT] vs [nonEXT]-[EXT] | 24 vs 24 | permuted t test | t=4.4987, df=46 | p<0.0001 |
| 4A-4 | rearing | [nonEXT]-[nonEXT] vs [nonEXT]-[EXT] | 24 vs 24 | permuted t test | t=2.6303, df=46 | not significant after FDR correction |
| 4B-4 | quiescence | [nonEXT]-[naive partner] vs [nonEXT]-[EXT] | 12 vs 24 | permuted t test | t=0.2404, df=34 | not significant after FDR correction |
| 4B-4 | mesh sniffing | [nonEXT]-[naive partner] vs [nonEXT]-[EXT] | 12 vs 24 | permuted t test | t=0.3844, df=34 | not significant after FDR correction |
| 4B-4 | cage exploration | [nonEXT]-[naive partner] vs [nonEXT]-[EXT] | 12 vs 24 | permuted t test | t=2.4674, df=34 | p=0.0201 |
| 4B-4 | rearing | [nonEXT]-[naive partner] vs [nonEXT]-[EXT] | 12 vs 24 | permuted t test | t=1.4348, df=34 | not significant after FDR correction |
| 4C-4 | quiescence | [nonEXT]-[unfamiliar rat] vs [nonEXT]-[EXT] | 12 vs 24 | permuted t test | t=0.8676, df=34 | not significant after FDR correction |
| 4C-4 | mesh sniffing | [nonEXT]-[unfamiliar rat] vs [nonEXT]-[EXT] | 12 vs 24 | permuted t test | t=0.22557, df=34 | not significant after FDR correction |
| 4C-4 | prosocial behavior | [nonEXT]-[unfamiliar rat] vs [nonEXT]-[EXT] | 12 vs 24 | permuted t test | t=0.4795, df=34 | not significant after FDR correction |
| 4C-4 | cage exploratiom | [nonEXT]-[unfamiliar rat] vs [nonEXT]-[EXT] | 12 vs 24 | permuted t test | t=2.2737, df=34 | p=0.0213 |
| 4C-4 | quiescence | [nonEXT]-[other strain rat] vs [nonEXT]-[EXT] | 12 vs 24 | permuted t test | t=0.8677, df=34 | not significant after FDR correction |
| 4C-4 | mesh sniffing | [nonEXT]-[other strain rat] vs [nonEXT]-[EXT] | 12 vs 24 | permuted t test | t=1.0300, df=34 | not significant after FDR correction |
| 4C-4 | cage exploration | [nonEXT]-[other strain rat] vs [nonEXT]-[EXT] | 12 vs 24 | permuted t test | T=2.2549, df=34 | not significant after FDR correction |
| 4C-4 | rearing | [nonEXT]-[other strain rat] vs [nonEXT]-[EXT] | 12 vs 24 | permuted t test | t=1.4431, df=34 | not significant after FDR correction |
| 4C-4 | quiescence | [nonEXT]-[unfamiliar rat] vs [nonEXT]-[other strain rat] | 12 vs 12 | permuted t test | t=0.1828, df=22 | not significant after FDR correction |
| 4C-4 | mesh sniffing | [nonEXT]-[unfamiliar rat] vs [nonEXT]-[other strain rat] | 12 vs 12 | permuted t test | t=1.5575, df=22 | not significant after FDR correction |
| 4C-4 | cage exploration | [nonEXT]-[unfamiliar rat] vs [nonEXT]-[other strain rat] | 12 vs 12 | permuted t test | t=0.0706, df=22 | not significant after FDR correction |
| 4C-4 | rearing | [nonEXT]-[unfamiliar rat] vs [nonEXT]-[other strain rat] | 12 vs 12 | permuted t test | t=0.9348, df=22 | not significant after FDR correction |
| 4C-5 | number of 22-kHz USVs | [nonEXT]-[unfamiliar rat] vs [nonEXT]-[EXT] | 12 vs 18 | permuted t test | t=2.2232, df=28 | not significant after FDR correction |
| 4C-5 | number of 22-kHz USVs | [nonEXT]-[other strain rat] vs [nonEXT]-[EXT | 12 vs 18 | permuted t test | t=1.0978, df=28 | not significant after FDR correction |
| 4C-5 | number of 50-kHz USVs | [nonEXT]-[other strain rat] vs [nonEXT]-[EXT] | 12 vs 18 | permuted t test | t=1.9279, df=28 | not significant after FDR correction |
| 4C-5 | number of 22-kHz USVs | [nonEXT]-[unfamiliar rat] vs [nonEXT]-[other strain rat] | 12 vs 12 | permuted t test | t=0.9623, df=22 | not significant after FDR correction |
| 4C-5 | number of 50-kHz USVs | [nonEXT]-[unfamiliar rat] vs [nonEXT]-[other strain rat] | 12 vs 12 | permuted t test | t=0.2620, df=22 | not significant after FDR correction |
| **Figure 5** | | | | | | |
| 5B | freezing | [nonEXT]-[EXT] **Test 2 vs** [nonEXT]-[EXT] **Test 3** | 12 vs 12 | permuted t test | t=4.2732, df=20 | **p=0.0047** |
| 5C | freezing | [nonEXT]-[EXT] **Test 2 vs** [nonEXT]-[EXT] **Test 3** | 12 vs 12 | mixed ANOVA | group, F(1, 330)= 15.1019  time, F(15, 330)=7.6464  group x time, F(15, 330)=3.8011 | **p=0.0008**  **p<0.0001**  **p<0.0001** |
| 5B | freezing | [nonEXT]-[EXT] **Test 1 vs** [nonEXT]-[EXT] **Test 3** | 12 vs 12 | permuted t test | t=0.2847, df=20 | not significant after FDR correction |
| 5C | freezing | [nonEXT]-[EXT] **Test 1 vs** [nonEXT]-[EXT] **Test 3** | 12 vs 12 | mixed ANOVA | group, F(1, 330)= 0.0282  time, F(15, 330)=7.2579  group x time, F(15, 330)=1.2742 | not significant after FDR correction  **p<0.0001**  not significant after FDR correction |
| 5B | freezing | [nonEXT] **Test 1** vs [nonEXT] **Test 2** | 11 vs 11 | permuted t test | t=4.2060, df=18 | **p=0.0079** |
| 5C | freezing | [nonEXT] **Test 1** vs [nonEXT] **Test 2** | 11 vs 11 | mixed ANOVA | group, F(1, 300)= 13.7190  time, F(15, 300)=16.0570  group x time, F(15, 300)=4.5385 | **p=0.0013**  **p<0.0001**  **p<0.0001** |
| 5B | freezing | [nonEXT] **Test 2** vs [nonEXT] **Test 3** | 11 vs 10 | permuted t test | t=2.9135, df=17 | **p=0.0077** |
| 5C | freezing | [nonEXT] **Test 2** vs [nonEXT] **Test 3** | 11 vs 11 | mixed ANOVA | group, F(1, 285)=10.5307  time, (F15, 285)=4.2803  group x time F(1, 285)=1.4207 | **p=0.0043**  **p<0.0001**  not significant after FDR correction |
| 5B | freezing | [nonEXT] **Test 1** vs [nonEXT] **Test 3** | 11 vs 11 | permuted t test | t=12.0473, df=17 | **p<0.0001** |
| 5C | freezing | [nonEXT] **Test 1** vs [nonEXT] **Test 3** | 11 vs 11 | mixed ANOVA | group, F(1, 285)=162.3087  time, (F15, 285)=8.3539  group x time F(1, 285)=2.6787 | **p<0.0001**  **p<0.0001**  **p=0.0008** |
| 5D | cage exploration | [nonEXT]-[EXT] vs [nonEXT] **Test 1** | 12 vs 11 | permuted t test | t=4.0913, df=19 | **p=0.0005** |
| 5D | rearing | [nonEXT]-[EXT] vs [nonEXT] **Test 1** | 12 vs 11 | permuted t test | t=1.0550, df=19 | not significant after FDR correction |
| 5D | cage exploration | [nonEXT]-[EXT] vs [nonEXT] **Test 2** | 12 vs 11 | permuted t test | t=0.5091, df=19 | not significant after FDR correction |
| 5D | rearing | [nonEXT]-[EXT] vs [nonEXT] **Test 2** | 12 vs 11 | permuted t test | t=1.8307, df=19 | not significant after FDR correction |
| 5D | cage exploration | [nonEXT]-[EXT] vs [nonEXT] **Test 3** | 12 vs 11 | permuted t test | t=1.0417,df=18 | not significant after FDR correction |
| 5D | rearing | [nonEXT]-[EXT] vs [nonEXT] Test 3 | 12 vs 11 | permuted t test | t=0.0106, df=18 | not significant after FDR correction |
| 5D | quiescence | [nonEXT]-[EXT] **Test 1** vs [nonEXT]-[EXT] **Test 2** | 12 vs 12 | permuted t test | t=2.5921, df=20 | **p=0.0271** |
| 5D | mesh sniffing | [nonEXT]-[EXT] **Test 1** vs [nonEXT]-[EXT] **Test 2** | 12 vs 12 | permuted t test | t=3.1750, df=20 | **p=0.0081** |
| 5D | cage exploration | [nonEXT]-[EXT] **Test 1** vs [nonEXT]-[EXT] **Test 2** | 12 vs 12 | permuted t test | t=0.9300, df=20 | not significant after FDR correction |
| 5D | rearing | [nonEXT]-[EXT] **Test 1 vs** [nonEXT]-[EXT] **Test 2** | 12 vs 12 | permuted t test | t=2.8480, df=20 | **p=0.0063** |
| 5D | quiescence | [nonEXT]-[EXT] **Test 2** vs [nonEXT]-[EXT] **Test 3** | 12 vs 12 | permuted t test | t=3.3477, df=20 | **p=0.0117** |
| 5D | mesh sniffing | [nonEXT]-[EXT] **Test 2 vs** [nonEXT]-[EXT] **Test 3** | 12 vs 12 | permuted t test | t=1.0915, df=20 | not significant after FDR correction |
| 5D | cage exploration | [nonEXT]-[EXT] **Test 2** vs [nonEXT]-[EXT] **Test 3** | 12 vs 12 | permuted t test | t=0.2426, df=20 | not significant after FDR correction |
| 5D | rearing | [nonEXT]-[EXT] **Test 2 vs** [nonEXT]-[EXT] **Test 3** | 12 vs 12 | permuted t test | t=2.5645 | **p=** **0.0065** |
| 5D | quiescence | [nonEXT]-[EXT] **Test 1** vs [nonEXT]-[EXT] **Test 3** | 12 vs 12 | permuted t test | t=1.0678, df=20 | not significant after FDR correction |
| 5D | mesh sniffing | [nonEXT]-[EXT] **Test 1 vs** [nonEXT]-[EXT] **Test 3** | 12 vs 12 | permuted t test | t=4.1643, df=20 | **p=0.0007** |
| 5D | cage exploration | [nonEXT]-[EXT] **Test 1 vs** [nonEXT]-[EXT] **Test 3** | 12 vs 12 | permuted t test | t=0.4172, df=20 | not significant after FDR correction |
| 5D | rearing | [nonEXT]-[EXT] **Test 1 vs** [nonEXT]-[EXT] **Test 3** | 12 vs 12 | permuted t test | t=0.7434, df=20 | not significant after FDR correction |
| 5D | quiescence | [nonEXT] Test 1 vs [nonEXT] Test 2 | 11 vs 11 | permuted t test | t=6.3443, df=18 | **p<0.0001** |
| 5D | mesh sniffing | [nonEXT] **Test 1** vs [nonEXT] **Test 2** | 11 vs 11 | permuted t test | t=2.4820, df=18 | **p<0.0001** |
| 5D | cage exploration | [nonEXT] **Test 1** vs [nonEXT] **Test 2** | 11 vs 11 | permuted t test | t=1.0655, df=18 | not significant after FDR correction |
| 5D | rearing | [nonEXT] **Test 1** vs [nonEXT] **Test 2** | 11 vs 11 | permuted t test | t=0.4567, df=18 | not significant after FDR correction |
| 5D | quiescence | [nonEXT] **Test 2** vs [nonEXT] **Test 3** | 11 vs 11 | permuted t test | t=3.3432, df=17 | **p=0.0039** |
| 5D | mesh sniffing | [nonEXT] **Test 2** vs [nonEXT] **Test 3** | 11 vs 11 | permuted t test | t=1.2560, df=17 | not significant after FDR correction |
| 5D | cage exploration | [nonEXT] **Test 2** vs [nonEXT] **Test 3** | 11 vs 11 | permuted t test | t=0.2131, df=17 | not significant after FDR correction |
| 5D | rearing | [nonEXT] **Test 2** vs [nonEXT] **Test 3** | 11 vs 11 | permuted t test | t=3.5795, df=17 | **p=0.0023** |
| 5D | quiescence | [nonEXT] **Test 1** vs [nonEXT] **Test 3** | 11 vs 11 | permuted t test | t=9.1132, df=17 | **p<0.0001** |
| 5D | mesh sniffing | [nonEXT] **Test 1** vs [nonEXT] **Test 3** | 11 vs 11 | permuted t test | t=6.5953, df=17 | **p<0.0001** |
| 5D | cage exploration | [nonEXT] **Test 1** vs [nonEXT] **Test 3** | 11 vs 11 | permuted t test | t=1.6916, df=17 | not significant after FDR correction |
| 5D | rearing | [nonEXT] **Test 1** vs [nonEXT] **Test 3** | 11 vs 11 | permuted t test | t=3.0535, df=17 | **p=0.0063** |
| 5E | number of 22 kHz USVs | [nonEXT]-[EXT] **Test 1 vs** [nonEXT]-[EXT] **Test 3** | 12 vs  8 | permuted t test | t=0.3607, df=18 | not significant after FDR correction |
| 5E | number of 55 kHz USVs | [nonEXT]-[EXT] **Test 1 vs** [nonEXT]-[EXT] **Test 3** | 12 vs  8 | permuted t test | t=3.4889, df=18 | **p<0.0001** |
| 5E | number of 22 kHz USVs | [nonEXT]-[EXT] **Test 2 vs** [nonEXT]-[EXT] **Test 3** | 9 vs  8 | permuted t test | t=4.6274, df=15 | **p=0.0005** |
| 5E | number of 55 kHz USVs | [nonEXT]-[EXT] **Test 2 vs** [nonEXT]-[EXT] **Test 3** | 9  vs  8 | permuted t test | t=0.0309, df=15 | not significant after FDR correction |
| 5E | number of 22 kHz USVs | [nonEXT] **Test 1** vs [nonEXT] **Test 2** | 11 vs 10 | permuted t test | t=3,8546, df=19 | **p=0.0019** |
| 5E | number of 50 kHz USVs | [nonEXT] **Test 1** vs [nonEXT] **Test 2** | 11 vs 10 | permuted t test | t=0.3923, df=19 | not significant after FDR correction |
| 5E | number of 22 kHz USVs | [nonEXT] **Test 1** vs [nonEXT] **Test 3** | 11  vs  9 | permuted t test | t=4.8574, df=18 | **p<0.0001** |
| 5E | number of 55 kHz USVs | [nonEXT] **Test 1** vs [nonEXT] **Test 3** | 11  vs  9 | permuted t test | t=0.1976, df=18 | not significant after FDR correction |
| 5E | number of 22 kHz USVs | [nonEXT] **Test 2** vs [nonEXT] **Test 3** | 10 vs  9 | permuted t test | t=0.9831, df=17 | not significant after FDR correction |
| 5E | number of 55 kHz USVs | [nonEXT] **Test 2** vs [nonEXT] **Test 3** | 10 vs  9 | permuted t test | t=0.5356, df=17 | not significant after FDR correction |
| **Figure 7** | | | | | | |
| 7B-4 | quiescence | vHip-PL vs Control | 10 vs 9 | permuted t test | t=0.2083, df=15 | not significant after FDR correction |
| 7B-4 | prosocial | vHip-PL vs Control | 10 vs 9 | permuted t test | t=0.7366, df=15 | not significant after FDR correction |
| 7B-4 | cage exploration | vHip-PL vs Control | 10 vs 9 | permuted t test | t=1.1334, df=15 | not significant after FDR correction |
| 7B-4 | rearing | vHip-PL vs Control | 10 vs 9 | permuted t test | t=1.3569, df=15 | not significant after FDR correction |
| 7B-4 | prosocial | ACC-CeA vs Control | 9 vs 9 | permuted t test | t=2.6113, df=14 | not significant after FDR correction |
| 7B-4 | quiescence | vHipp-PL vs ACC-CeA |  | permuted t test | t=4.1037, df=15 | **p=0.0029** |
| 7B-4 | mesh sniffing | vHipp-PL vs ACC-CeA |  | permuted t test | t=2.2852, df=15 | **p=0.0321** |
| 7B-4 | prosocial | vHipp-PL vs ACC-CeA |  | permuted t test | t=2.1861, df=15 | not significant after FDR correction |
| 7B-4 | cage exploration | vHipp-PL vs ACC-CeA |  | permuted t test | t=5.0920, df=15 | **p<0.0001** |
| 7B-4 | rearing | vHipp-PL vs ACC-CeA |  | permuted t test | t=4.7463, df=15 | **p=0.0007** |
