# Supplementary material for: Social buffering diminishes fear response but does not equal improved fear extinction
Source: Cereb Cortex. 2022 Oct 11;33(8):5007–24. doi: 10.1093/cercor/bhac395 (PMC10110450; doi:10.1093/cercor/bhac395)
Supplement: suplementary_materials_EK_bhac395 [file suplementary_materials_ek_bhac395.docx]

**Materials and methods**

**Animals**

For axonal tracing experiments, subjects were 6 male c-Fos-PSD95 Venus-Arc rats (Knapska et al. 2012). The rats (300–400 g at the beginning of the experiment) were bred at the Nencki Institute Animal House or Faculty of Biology Animal House (University of Warsaw). Rats were housed and habituated in the same way as Wistar rats.

**Functional tract tracing**

**Surgical procedure**

One week prior to behavioral training, rats received bilateral intracranial injections of the anterograde axonal transport tracers tetramethylrhodamine (FluoroRuby, FR) and PHA-L Alexa Fluor 647 conjugate (Invitrogen; Molecular Probes) into the BL and vHIP. The sides of the injection and the type of the tracer were counterbalanced between the brain structures.

All surgical instruments were sterilized before surgery. Rats were anesthetized with isoflurane (Aerane, 5% induction, 1% for maintenance), subcutaneously injected with an analgesic (Butorfanol, 1 mg/kg) and placed into the stereotaxic apparatus (David Kopf Instruments). Eyes were moistened with ocular lubricant and the scalp was shaved. Next the scalp was disinfected with 70% (vol/vol) alcohol, incised, and skin was retracted. Four small burr holes were drilled to allow for a 1 µl NanoFil syringe needle (World Precision Instruments) to be lowered into the desired part of the brain. The coordinates used were: BL [anteroposterior (AP): -2.2; mediolateral (ML): ±4.8; dorsoventral (DV): −8.6], vHIP [AP: −5.3, ML: ±5.5, DV: −7.0].

Tracers: FR [10% (wt/vol) solution in distilled water] and PHA-L [2.5% (wt/vol) solution in 0.1 M sodium PBS, pH 7.4] were delivered into the BL and vHIP with a Nanofil syringe (MicroSyringe Pump; World Precision Instruments; (500 nl per hemisphere, 100 nl/min); the needle remained in place for another 5 min to allow for the diffusion of the tracer). After the injection, the incision was sutured, and the animals were administered an analgesic (Tolfedine; 4 mg/kg; s.c.) and an antibiotic (Baytril; 4 mg/kg; s.c). To avoid dehydration the animals were given 1 ml of 0.95% NaCl/100 g of body weight by s.c. injection. The rats were kept on a heating pad until they recovered from anesthesia before returning to their home cages. The animals were allowed 7 days of postoperative recovery.

**Fluorescent immunostaining for GFP**

GFP fluorescent staining was performed on free-floating sections. The sections were washed with PBS with 0.3% Triton X-100 (PBST), blocked with 10% (vol/vol) normal goat serum in PBST, and incubated overnight at 4°C with anti-GFP rabbit antibody (Invitrogen) diluted with 1% normal goat serum (NGS) in PBST. The next day, sections were rinsed with PBST before 1 h incubation at room temperature with a secondary antibody conjugated to Alexa Fluor 488 (1:500; Invitrogen). After several washes, the sections were mounted onto glass slides, overlaid with the Fluoromount G Medium, and covered with a glass coverslip.

**Analysis of functional tract tracing**

The potential contacts between PHA-L- and FR-labeled fibers (afferent terminals) and the Venus-positive neurons were estimated as numbers of voxels for axonal varicosities located in the close proximity to Venus-positive neurons [see (Knapska et al. 2012) for details]. Then, the ratios of vHIPP/BL projections (measured in voxels) were calculated for all Venus-positive neurons in a single scan image for the PL and averaged between two images for each rat. In another analysis, the ratios of vHIPP/BL projections were calculated for single Venus-positive neurons within the PL and represented as the percentage of neurons with a certain BL/vHIP input ratio onto the active cells. Differences between the rate of afferent projections from the BL and vHIP onto activated neurons in the PL for the rats subjected to fear extinction alone ([nonEXT]) and with a partner ([nonEXT]-[EXT]) were compared. Cumulative frequency distributions for each group were made with the same fourteen arbitrary chosen intervals (0-0.19, 0.2-0.39, 0.4-0.59, 0.6-0.79, 0.8-0.99, 1.0-1.19, 1.2-1.39, 1.4-1.59, 1.6-1.79, 1.8-1.99, 2.0-2.19, 2.2-2.39, 2.4-2.59, and >2.6) used for each distribution. The number of cells used for the cumulative frequency distributions comparison: the rats tested alone - 126, the rats tested together - 129. The test focused on the largest of the observed deviations. To analyze the difference in projections distributions Kolmogorov-Smirnov two-sample test was used.

**Results**

Functional tracing

We traced functional projections, i.e., projections on activated neurons in the PL. We compared projections from the vHIP and BL (cf. Szadzinska et al., 2021). To this end, we used transgenic rats expressing a PSD-95:Venus fusion protein under the control of a c-fos promoter injected with anterograde tracers into the vHIP and the BL (Knapska et al. 2012). We found that activated cells in the PL of rats subjected to fear extinction in pairs receive more prominent input from the vHIP than from the BL (Figure S1, t test, t=3.758, df=4, p=0.0198; Figure S1, Kolmogorov–Smirnov two-sample test, Dmax=1.0, ks=2.65, p=0.000002).


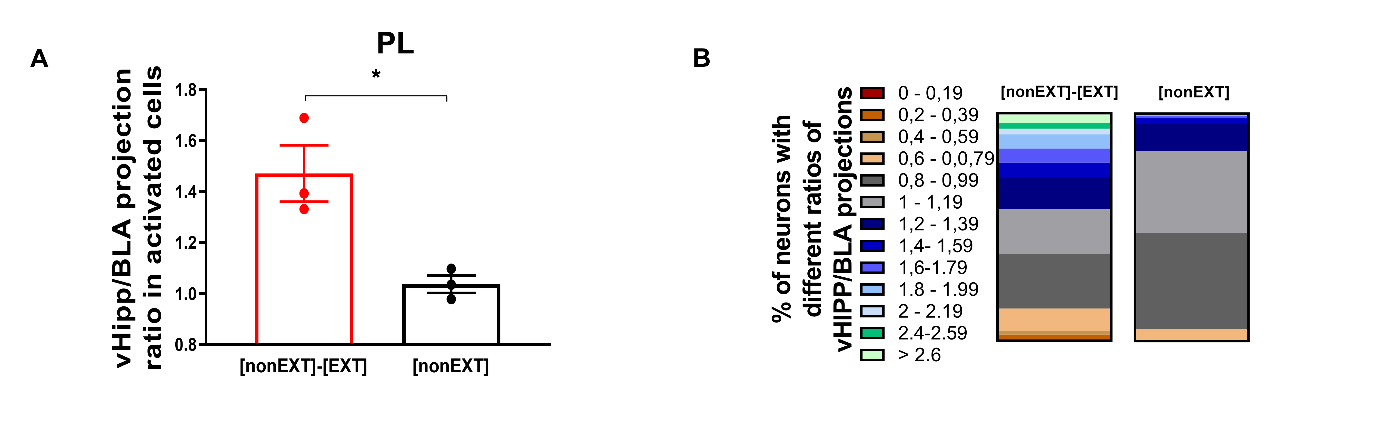


**Figure S1. Social buffering activates more PL neurons receiving projections from the vHIPP than from the BL.**

(A). The proportion of projections from the vHIPP and BL on active neurons in the PL in the accompanied and unaccompanied extinction groups. Active neurons expressing PSD-95:Venus fusion protein under the control of a c-fos promoter which receives inputs from the vHIP and the BL were analyzed. Cells activated in the PL by the accompanied ([nonEXT]-[EXT], red, n=3) extinction had a higher proportion of vHIP inputs than cells activated by unaccompanied ([nonEXT], black, n=3) extinction. (B) Percentage of neurons with different ratios of vHIP/BL projections plotted for the accompanied ([nonEXT]-[EXT], n=3) and unaccompanied ([nonEXT], n=3) extinction groups. The graphs show means ± SEM, * p <0.05.

**References**:

Knapska E, Macias M, Mikosz M, Nowak A, Owczarek D, Wawrzyniak M, Pieprzyk M, Cymerman IA, Werka T, Sheng M, et al. 2012. Functional anatomy of neural circuits regulating fear and extinction. Proc Natl Acad Sci U S A. 109:17093–17098.
